# Genetic Association of the Renin-Angiotensin-Aldosterone System with hypertension among the Malays and their adaptation to climate change

**DOI:** 10.1371/journal.pone.0346614

**Published:** 2026-04-15

**Authors:** Nur Hasnah Maamor, Alvin Cengnata, Yuwen Pan, Johan Rizal Ismail, Khasnur Abd Malek, Gordon H. Williams, Khalid Yusoff, Hoh Boon-Peng

**Affiliations:** 1 Faculty of Medicine and Health Sciences, UCSI University, UCSI Hospital, Port Dickson, Negeri Sembilan, Malaysia; 2 Evidence Based Healthcare Sector, National Institute of Health (NIH), Shah Alam, Selangor, Malaysia; 3 Faculty of Applied Science, UCSI University, Wilayah Persekutuan, Kuala Lumpur, Malaysia; 4 Key Laboratory of Computational Biology, Shanghai Institute of Nutrition and Health, University of Chinese Academy of Sciences, Chinese Academy of Sciences, Shanghai, China; 5 Cardiology Unit, Prince Court Medical Centre, Jalan Kia Peng, Kuala Lumpur, Malaysia; 6 UiTM Private Specialist Centre, Jalan Hospital, Sungai Buloh, Selangor, Malaysia; 7 MAA Medicare Cardiac Diagnostic Centre, Titiwangsa Central, Kuala Lumpur, Malaysia; 8 Department of Primary Care Medicine, Faculty of Medicine, Universiti Teknologi MARA, Sungai Buloh Campus, Selangor Branch, Jalan Hospital, Sungai Buloh, Selangor, Malaysia; 9 Division of Endocrinology, Diabetes, Hypertension, Brigham and Women’s, Harvard Medical School, Boston, Massachusetts, United States of America; 10 Division of Applied Biomedical Sciences and Biotechnology, School of Health Sciences, IMU University, Bukit Jalil, Kuala Lumpur, Malaysia; UCMI: University College MAIWP International, MALAYSIA

## Abstract

Hypertension (HT) is a ‘by-product’ to the forces of natural selection against environmental drift and salt availability, therefore contributed to differential HT susceptibility. This study provides further supporting evidence through: (i) associating three salt-sensitive related candidate gene variants, to the susceptibility of HT among the Malays from Peninsular Malaysia with a detail genotype-phenotype evaluation; (ii) comparing the blood pressure and the frequency spectrums of these variants across global populations; (iii) correlating them with the geographical coordinates and BP of the respective populations, and evaluating the presence of local adaptation in these candidate variants. We tested the genetic association of six SNPs underlying *CYP11B2*, *AGT* and *ADRB2* in 918 normotensives and hypertensives Malays, men and women. *CYP11B2* and *ADRB2* were associated with elevated BP in males and females, respectively. Evaluation of these gene variations across 38 populations residing in different latitudinal clines revealed strong correlations between *CYP11B2*, *AGT* and latitudinal coordinates; whilst *ADRB2* to a weaker extent. Tajima’s D analyses suggested a non-neutral evolution on *CYP11B2*, which suggested a modest putative signals of local adaptation. In summary, we complement the notion that effective pharmacogenetic marker(s) to predict responsiveness of anti-HT medication requires comprehensive characterization of population genetics and HT phenotypes.

## Introduction

Hypertension (HT) affects 1.2 billion people, worldwide. Hypertensive individuals are predisposed to various cardiovascular complications including heart failure, stroke, renal failure and peripheral vascular disease, hence a major factor contributing to global morbidity and mortality. There is a complex interplay between genetics, environment and lifestyle that confers an individual’s susceptibility to HT [1–3]. The prevalence of HT varies substantially across populations of different ancestries [1–4] and may present with different phenotypes: (i) essential HT without an identifiable etiology vs secondary HT resulted from a known underlying cause; (ii) ~60% of essential HT have significant elevations of blood pressure (BP) in response to a change in salt intake hence salt-sensitive HT (SS-HT); and (iii) 55% of the SS-HT are low-renin while the remaining present with normal-renin levels (non-modulation) [5].

From the evolutionary perspective, essential HT is arguably a ‘by-product’ to the forces of natural selection, possibly against environmental drift and salt availability [6]. It is believed that populations in the tropics experienced heat dissipation via increased sweating. While this is an efficient way for heat loss, it has unavoidable, and potentially lethal, side effect of salt loss. Additionally, salt availability was limited in many tropical areas. Thus, survivors in this climate have adapted to be more efficient in retaining sodium, thereby avoiding electrolyte imbalance and death.

Renin-Angiotensin-Aldosterone System (RAAS) is a major BP regulator via its role in controlling sodium homeostasis and vascular reactivity. The initiating step in this system is angiotensinogen (AGT) that serves as a substrate for renin which cleaves off angiotensin I (Ang I). Ang I serves as the substrate for angiotensin converting enzyme (ACE) converting it to angiotensin II (Ang II). Ang II modulates sodium homeostasis by: (i) regulating renal blood flow (RBF); and (ii) stimulating the secretion of aldosterone (ALDO). Both of these effects modify renal sodium loss or retention. Defects in these normal regulatory functions can increase sodium and water reabsorption, expand extracellular fluid volume and result in salt-sensitive BP (SS-BP). The last step of ALDO biosynthesis is aldosterone synthase encoded by the *CYP11B2* gene. Notably, this step is highly regulated by sodium intake: increasing salt intake reduces its activity and eventually its production with a *vice versa* effect when salt intake is increased. Specifically, genetic association of the selected variants *AGT* (rs699 and rs5051), *CYP11B2* (rs1799998 and rs10087214); and *ADRB2* (rs1042713 and rs1042714) (**S1 Table**) with SS-HT have been extensively investigated [3,5,7–12]. The *AGT* and *CYP11B2* variants have been associated with non-modulation SS-HT [5,13,14]; whereas the *ADRB2* variants are associated with low renin SS-HT [5,15]. Owing to their known attributions to HT intermediate phenotypes, these variants thus potentially serve as the pharmacogenetic markers to predict the response against anti-hypertensive medications, such as angiotensin receptor blocker, beta-blocker, and mineralocorticoid receptor antagonist.

What is puzzling, despites the vital roles on modulating sodium homeostasis hence BP regulation, classical case-control studies and linkage analysis between HT and the selected variants of these genes yielded equivocal and inconsistent findings in populations from various continents [9,13,16–19], although the Southeast Asian natives like the Malays from Peninsular Malaysia have not been systematically evaluated. The selected variants of interest exhibited substantial variation across populations of different ancestries from different continents. Selection pressures was thought to be the reason to the *AGT* and *ADRB2* diversity [6], however evidence supporting natural selection in *CYP11B2*, if any, for its identified functional variants has not been reported.

These collective rationales lead us to hypothesize that (i) the genetic variation of the candidate genes that regulate RAAS (namely, the *AGT* and *CYP11B2*) confers to the variability of mean BP, hence susceptibility to HT, among the Malays from Peninsular Malaysia; (ii) the variability of mean BP regulated by RAAS, hence susceptibility to HT across populations from different continents, may be a consequence of salt and heat adaptation; (iii) *AGT* and *CYP11B2* therefore may have co-evolved under the same selection pressures owing to their vital roles in RAAS, we anticipate; but not *ADRB2*.

This study provides further supporting evidence to the hypotheses through: (i) a more detail genotype-phenotype evaluation by performing genetic association of the selected variants of interest: *AGT* (rs699 and rs5051), *CYP11B2* (rs1799998 and rs10087214); and *ADRB2* (rs1042713 and rs1042714), to the susceptibility of HT among the Malays from Peninsular Malaysia; (ii) comparing the mean BP and the frequency spectrums of these variants across 38 populations globally, and correlating them with the geographical coordinates of the respective populations; and (iii) providing a supporting evidence to the putative signal of local adaptation for *CYP11B2*.

## Materials and methods

### Recruitment

A total of 418 normotensive (NT) participants and 500 HT patients were recruited between August 2016 and August 2018. They were derived from the community based epidemiological study REsponDing to IncreaSing CardiOVascular disEase pRevalence (REDISCOVER) Study [20]. Informed and written consent was obtained from each participating individuals. Ethical approval was obtained from Universiti Teknologi MARA Research and Ethics Committee (Ref no: 600-RMI (5/1/6)).

NT was defined as: (i) systolic BP (SBP) and diastolic BP (DBP) ≤130/80 mmHg during the time of recruitment; (ii) no history of anti-HT medication. HT was defined as: (i) SBP ≥ 140 mmHg or/and DBP ≥ 90 mmHg; (ii) subject on anti-HT medication during recruitment; or (iii) subject with previous history of HT. The participants were recorded to have normal dietary salt intake during the recruitment of study, therefore their salt-sensitivity characteristics was not assessed.

The recruited subjects were age between 35–70 years old and Malay ethnicity. Subjects were excluded if: (i) no consent was given; (ii) pregnant females; (iii) Body Mass Index (BMI) >32; (iv) current excessive alcohol consumption; (v) diagnosed to have secondary HT; (vi) diagnosed with: diabetes mellitus, primary cardiac valvular disease, active myocarditis, history of myocarditis, hypertrophic or restrictive cardiomyopathy, pericardial disease, arrhythmia, or primary hepatic, renal, neurological, pulmonary or endocrine diseases.

### DNA extraction and genotyping

Four millilitres of peripheral blood was withdrawn from the participants. DNA was extracted using QIAGEN Blood Mini DNA Extraction kit (Qiagen, Hilden, Germany) following the manufacturer’s instruction.

Genotyping was carried out either by Agena Mass Array (Agena Bioscience) or quantitative-PCR allelic discrimination (Applied Biosystem). Additional data previously genotyped on 121 HT samples with SNP array (Illumina Inc.) were also included as previously reported [21]. The same inclusion and exclusion criteria were applied on these samples.

### Global population study

The variants frequencies of 38 selected global populations were retrieved from the 1000 Genomes Project (https://www.ncbi.nlm.nih.gov/variation/tools/1000genomes/), Human Genome Diversity Project (HGDP) panel [22]; Singapore Genome Variation Project (SGVP) [23], and the genotyping data for the indigenous Negrito populations from Peninsular Malaysia and North Borneo populations [24,25]. Any genotype and allele frequencies not reported from one of the resources above, were retrieved from the PGG.SNV database (https://www.pggsnv.org/index.html) [26]. We characterized the distribution of the allele and genotype frequencies of the selected populations and correlated them with the geographical latitude of the respective populations.

### Statistical analysis

Demographic data were analyzed using Fisher exact test or Student independent T-test.

Genetic association between the variants of interest and HT was performed using Fisher exact test. Statistical adjustment was applied using logistics regression (by adjusting the confounding covariates including age, sex, BMI and anti-HT medications). The significance of the risk alleles and genotypes in altering BP were tested with student’s T-test. In this study, SBP was taken as the quantitative BP measurement.

Pearson’s correlation coefficient was computed to assess the correlation between the risk alleles and genotypes frequencies, versus distance of geographic latitudes from equator. P < 0.05 was considered to be significant.

### Analysis of Tajima’s *D*

Tajima’s *D* statistic was computed to assess deviations from neutrality in the genetic dataset following the protocol reported by [27]. The analysis was carried out using 100 Malay genome sequence randomly selected from publicly available datasets [28,29]. Prior to the analysis, the datasets were imputed using the Southeast Asia specific reference panel [29]. In brief, the SG10K dataset were cross-imputed with GA100K; followed by GA100K with SG10K serving as a reference panel. The datasets were then merged, followed by a cross-imputation with the Orang Asli and Papuan genome sequencing datasets obtained from publicly available datasets, or published datasets with permission from respective authors [29]. Validation of the imputation reference panel and “cross-imputation” approach revealed 40% genotyping recall rate as opposed to∼16% and ∼30% using Trans-Omics for Precision Medicine (TOPMed) and 1KGP reference panels, and 2%–5% lower non-disconcordance rate, collectively proven the reliability of the imputed datasets. The imputed data yielded ∼113 millions of SNPs. SNPs were filtered using an INFO SCORE ≥0.8 provided by IMPUTE5.

Chromosome-wide genetic diversity was estimated within sliding windows of 5 kb in length. Tajima’s *D*, Fu and Li’s *D* and Fu and Li’s *S* statistics were calculated [30]. The empirical *P* value was estimated by ranking the statistical values of *CYP11B2* and *AGT* (as benchmark) among all the sliding windows on protein-coding genes across the chromosome 8 (where *CYP11B2* is located) and chromosome 1 (where *AGT* is located).

Negative Tajima’s D values indicate an excess of low-frequency variants, suggesting population expansion or positive selection, whereas positive values reflect a deficiency of rare variants, implying balancing selection or population bottlenecks.

## Results

### Anthropometric and clinical parameters

The HT and NT groups were significantly different in all parameters except glucose level, even though stringent exclusion criteria were applied (**Table 1**). HT were on average older, had higher BMI, waist circumference and TG level, but lower levels of blood glucose, HDL, LDL and TC.

**Table 1:** Anthropometric and clinical characteristics (mean ± s.e.m.) of all studied subjects.

	All			Female			Male		
Parameters	HT (N = 500)	NT (N = 418)	p-value	HT (N = 246)	NT (N = 239)	p-value	HT (N = 254)	NT (N = 179)	p-value
**Gender:**					–	–	–	–	–
**Male**	254	179	0.017*	–					
**Female**	246	239							
**BMI (kg/m**^**2**^)	25.6 ± 2.7	24.0 ± 3.2	<0.001*	54.2 ± 8.8	48.7 ± 7.8	<0.001*	56.1 ± 8.4	51.0 ± 8.0	<0.001*
**Age (years)**	55.2 ± 8.6	49.7 ± 8.6	<0.001*	25.7 ± 3.0	24.1 ± 3.3	<0.001*	25.6 ± 2.5	23.8 ± 3.0	<0.001*
**Systolic blood pressure (mmHg)**	149.4 ± 17.5	115.8 ± 7.5	<0.001*	147.5 ± 16.7	115.4 ± 7.4	<0.001*	151.2 ± 18.0	116.5 ± 7.7	<0.001*
**Diastolic blood pressure (mmHg)**	86.8 ± 10.4	71.8 ± 6.5	<0.001*	85.8 ± 11.2	72.1 ± 6.6	<0.001*	87.9 ± 9.4	71.5 ± 6.3	<0.001*
**Mean Arterial pressure (mmHg)**	107.7 ± 11.1	86.5 ± 6.0	<0.001*	106.3 ± 11.3	86.5 ± 6.0	<0.001*	109.0 ± 10.8	86.5 ± 6.0	<0.001*
**Waist circumferences (cm)**	85.9 ± 13.8	79.9 ± 11.6	<0.001*	82.3 ± 15.4	77.0 ± 11.4	<0.001*	89.4 ± 10.9	83.6 ± 10.7	<0.001*
**Glucose (mmol/L)**	4.7 ± 1.5	4.8 ± 0.6	0.060	4.5 ± 1.6	4.8 ± 0.6	0.024*	4.8 ± 1.4	4.9 ± 0.7	0.573
**High density lipid (HDL) (mg/dL)**	1.2 ± 0.5	1.3 ± 0.3	0.002*	1.3 ± 0.6	1.4 ± 0.3	0.059	1.0 ± 0.3	1.1 ± 0.2	0.102
**Low density lipid (LDL) (mg/dL)**	3.3 ± 1.3	3.8 ± 0.9	<0.001*	3.2 ± 1.4	3.7 ± 0.9	<0.001*	3.5 ± 1.3	3.9 ± 0.9	<0.001*
**Total Cholesterol (TC) (mg/dL)**	5.2 ± 1.7	5.8 ± 1.0	<0.001*	5.1 ± 1.8	5.7 ± 1.0	<0.001*	5.4 ± 1.6	5.8 ± 1.1	0.001*
**Triglyceride (TG) (mg/dL)**	1.5 ± 0.9	1.4 ± 0.7	0.049*	1.4 ± 0.8	1.3 ± 0.7	0.472	1.7 ± 1.0	1.6 ± 0.7	0.175

‘-‘, not applicable; HT, hypertension; NT, normotension.

### Genetic association with HT

Early studies documented that sex difference underlies the non-modulation HT intermediate phenotype (associated with *AGT* variants), possibly due to the contribution of female sex hormones to protection against genotypic predisposition in premenopausal females [31]. Further, the expression of non-modulating phenotype of HT in females was approximately half of that in males [14,32–34], and they had greater SS-BP than males owing to altered ALDO production [35]. Therefore, we performed the genetic association analysis with HT by sex.

Logistic regression (adjusted for confounding covariates including age, BMI, blood glucose, lipid profiles and anti-HT medications) found no significant association between the *AGT* and *ADRB2* SNPs and HT; except *CYP11B2*, with genotype distributions for both variants differed significantly between HT and NT (P_rs10087214_ = 0.015, and P_rs1799998_ = 0.038; **Table 2**). Specifically, the heterozygous GA genotype was more frequent among HT (38% vs 30% in NT), whereas the homozygous genotypes showed smaller differences. This trend suggests a possible of the A allele being the risk allele risk. The association remained when only males were analyzed (P = 0.034).

**Table 2:** Association analysis of the *CYP11B2* genetic variants with HT patients. Statistical adjustment was applied using logistics regression (by adjusting the confounding covariates including age, sex, BMI and history of anti-hypertension medication).

Gene	rsID#		Female			Male			All		
			HT	NT	p-value	HT	NT	p-value	HT	NT	p-value
			N = 246	N = 236	(LR)	N = 190	N = 178	(LR)	N = 498	N = 415	(LR)
***AGT***	rs699	**Genotype**									
		AA	0.02 (6)	0.03 (8)	0.730	0.02 (5)	0.05 (9)	0.212	0.02 (11)	0.04 (17)	0.253
		AG	0.29 (70)	0.26 (62)	(0.202)	0.29 (72)	0.29 (51)	(0.331)	0.28 (142)	0.27 (113)	(0.720)
		GG	0.69 (170)	0.71 (166)		0.69 (175)	0.66 (119)		0.68 (345)	0.69 (285)	
		**Allele**	**N = 492**	**N = 472**		**N = 504**	**N = 358**		**N = 996**	**N = 830**	
		A	0.18 (87)	0.17 (78)	0.633	0.16 (82)	0.19 (69)	0.535	0.17 (164)	0.18 (147)	0.492
		G	0.82 (405)	0.83 (394)	(0.724)	0.84 (422)	0.81 (289)	(0.654)	0.83 (832)	0.82 (683)	(0.992)
	rs5051	**Genotype**	**N = 242**	**N = 238**		**N = 233**	**N = 179**		**N = 498**	**N = 415**	
		TT	0.70 (170)	0.71 (170)	0.402	0.70 (164)	0.69 (123)	0.263	0.70 (335)	0.70 (293)	0.122
		TC	0.28 (68)	0.25 (60)	(0.196)	0.28 (65)	0.26 (48)	(0.388)	0.28 (133)	0.26 (108)	(0.757)
		CC	0.02 (4)	0.04 (8)		0.02 (4)	0.05 (8)		0.02 (8)	0.04 (16)	
		**Allele**	**N = 492**	**N = 476**		**N = 466**	**N = 358**		**N = 950**	**N = 417**	
		T	0.84 (411)	0.84 (399)	0.904	0.84 (393)	0.82 (292)	0.727	0.84 (801)	0.83 (694)	0.562
		C	0.16 (81)	0.16 (77)	(0.675)	0.16 (73)	0.18 (58)	(0.666)	0.16 (149)	0.17 (140)	(0.881)
***CYP11B2***	rs1799998	**Genotype**	**N = 237**	**N = 238**		**N = 190**	**N = 178**		**N = 427**	**N = 416**	
		GG	0.06 (15)	0.09 (21)	0.174	0.08 (16)	0.09 (16)	0.124	0.07 (31)	0.08 (37)	0.028*
		GA	0.41 (96)	0.33 (78)	(0.165)	0.41 (77)	0.30 (54)	(0.05)	0.41 (173)	0.32 (132)	(0.038)*
		AA	0.53 (126)	0.58 (139)		0.51 (97)	0.61 (108)		0.52 (223)	0.60 (247)	
		**Allele**	**N = 474**	**N = 476**		**N = 380**	**N = 340**		**N = 854**	**N = 832**	
		G	0.27 (126)	0.25 (120)	0.855	0.28 (109)	0.26 (87)	0.351	0.27 (235)	0.24 (205)	0.179
		A	0.73 (348)	0.75 (356)	(0.558)	0.72 (271)	0.74 (253)	(0.462)	0.73 (619)	0.76 (627)	(0.763)
	rs10087214	**Genotype**	**N = 246**	**N = 237**		**N = 252**	**N = 178**		**N = 498**	**N = 415**	
		GG	0.56 (138)	0.62 (146)	0.161	0.56 (140)	0.63 (112)	0.151	0.56 (278)	0.62 (258)	0.027*
		GA	0.39 (95)	0.31 (73)	(0.158)	0.38 (96)	0.29 (52)	(0.034)*	0.38 (191)	0.30 (125)	(0.015)*
		AA	0.05 (13)	0.07 (18)		0.06 (16)	0.08 (14)		0.06 (29)	0.08 (32)	
		**Allele**	**N = 492**	**N = 474**		**N = 504**	**N = 358**		**N = 996**	**N = 830**	
		G	0.75 (371)	0.77 (365)	0.957	0.75 (376)	0.75 (270)	0.785	0.75 (747)	0.77 (641)	0.294
		A	0.25 (121)	0.23 (109)	(0.686)	0.25 (128)	0.25 (88)	(0.666)	0.25 (249)	0.23 (189)	(0.928)
***ADRB2***	rs1042713	**Genotype**	**N = 246**	**N = 238**		**N = 252**	**N = 178**		**(N = 498)**	**(N = 416)**	
		GG	0.31 (75)	0.32 (77)	0.892	0.27 (69)	0.24 (42)		0.29 (144)	0.29 (119)	0.91
		GA	0.45 (112)	0.44 (104)	(0.129)	0.53 (133)	0.55 (97)	0.662	0.49 (245)	0.48 (201)	(0.443)
		AA	0.24 (59)	0.24 (57)		0.20 (50)	0.21 (39)	(0.506)	0.22 (109)	0.23 (96)	
		**Allele**	**N = 492**	**N = 476**		**N = 504**	**N = 358**		**N = 996**	**N = 832**	
		G	0.53 (262)	0.54 (258)	0.542	0.54 (271)	0.52 (186)	0.599	0.54 (533)	0.53 (439)	0.782
		A	0.47 (230)	0.46 (218)	(0.524)	0.46 (233)	0.48 (172)	(0.879)	0.46 (463)	0.47 (393)	(0.605)
	rs1042714	**Genotype**	**N = 242**	**N = 238**		**N = 216**	**N = 178**		**N = 458**	**N = 416**	
		CC	0.83 (201)	0.84 (200)	0.051	0.85 (183)	0.83 (148)	0.399	0.83 (384)	0.84 (348)	0.741
		CG	0.17 (41)	0.14 (33)	(0.400)	0.13 (29)	0.16 (29)	(0.060)	0.15 (70)	0.15 (62)	(0.149)
		GG	0.00 (0)	0.02 (5)		0.02 (4)	0.01 (1)		0.02 (4)	0.01 (6)	
		**Allele**	**N = 484**	**N = 476**		**N = 432**	**N = 350**		**N = 916**	**N = 832**	
		C	0.92 (443)	0.92 (433)	0.888	0.92 (395)	0.91 (321)	0.889	0.91 (838)	0.91 (758)	0.779
		G	0.08 (41)	0.08 (43)	(0.879)	0.08 (37)	0.09 (29)	(0.681)	0.09 (78)	0.09 (74)	(0.654)

P values for logistic regression in parentheses. HT, hypertension; NT, normotension; LR, logistic regression.

Further analysis suggested no significant association between the haplotypes of all candidate genes (**S2** **and** **S3 Tables**). The SNPs for *AGT* and *CYP11B2* are in strong linkage disequilibrium (LD) in the Malay population (D’ = 0.959, r^2^ = 0.837 for *AGT*; and D’ = 0.994, r^2^ = 0.89 for *CYP11B2*), but not for *ADRB2* (D’ = 0.913, r^2^ = 0.070) (**S1 Fig**). Owing to the strong LD on the *AGT* and *CYP11B2* variants, the following text shall denote the risk variants for rs699-G, rs5051-T as ‘*AGT* genotype’ or ‘*AGT* allele’; and rs1799998-G and rs10087214-A as ‘*CYP11B2* genotype’ or ‘*CYP11B2* allele’, and rs1042714-G as the ‘*ADRB2* genotype’ or ‘*ADRB2* allele’, unless otherwise stated.

When we excluded samples with anti-HT medication, the anthropometric parameters and genotype distributions remained broadly similar (**S4** **and** **S5 Tables**). However, the association signals found in *CYP11B2* was no longer statistically significant after adjustment, likely reflecting the reduced sample size and the small variant effect sizes, thence consequent loss of statistical power. Importantly, the direction of effect was consistent with the overall analysis, suggesting that while the evidence is weaker in this subset, the underlying trend remains.

### Genetic association with BP alteration

When assessing the significance of these variants in altering BP among the HT, only hypertensive individuals who had no previous record of anti-HT medication during recruitment were recruited in this analysis (N = 313) (**S6 Table**).

**Fig 1:**
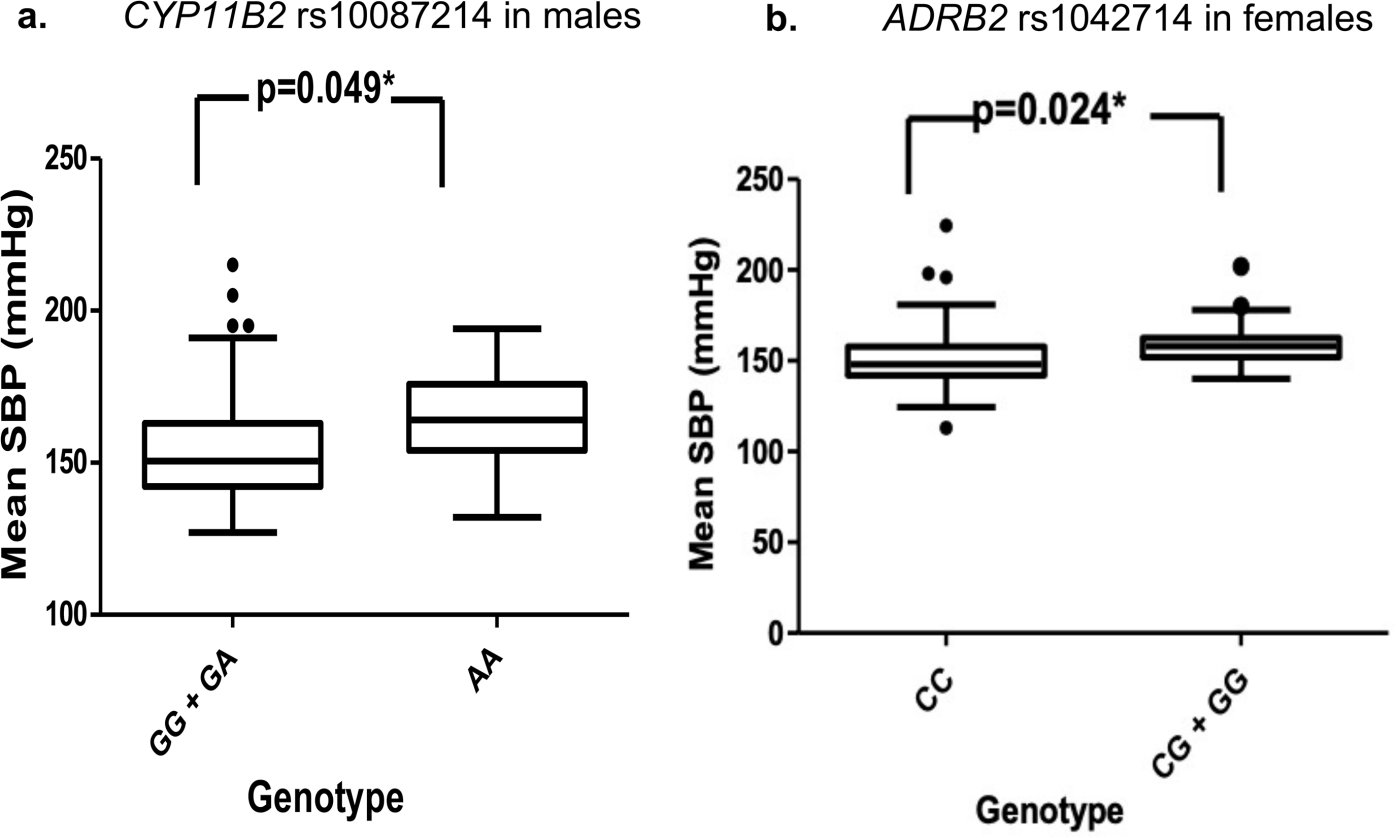
Effect of genotypes to the changes of SBP. (a) *CYP11B2* rs10087214-AA significantly elevated MAP in HT males; (b) *ADRB2* rs1042714-CC had lower SBP in females.

*AGT* showed no significant elevated BP, both in males and females (**S7 Table**); whereas HT males with *CYP11B2* rs1008721-AA genotype exhibited modestly higher BP (SBP, DBP, MAP) than other genotypes (P_SBP_ = 0.049, P_DBP_ = 0.024, and P_MAP_ = 0.013, respectively) (**Fig 1**; **S2 Fig**). *ADRB2* risk allele carriers (GG and CG) had higher SBP compared to the CC genotypes (p = 0.032), notably the females (P = 0.024) (**Fig 1**).

Further, HT males carrying the *CYP11B2* ‘G-A’ haplotype elevated BP as opposed to those who carried other haplotypes (P_SBP_ = 0.015, P_DBP_ = 0.047, and P_MAP_ = 0.029); whereas for male HT ‘GG-AA’ risk diplotype carrier, although not statistically significant, their SBP exhibited a trend on average 10 mmHg elevated SBP (P = 0.071); whereas the DBP and MAP were respectively, on average 7 mmHg and 8 mmHg (P_DBP_ = 0.025, and P_MAP_ = 0.018) higher than the rest (**S8 Table**). We note that the number of GG-AA male carrier is small therefore the results should be interpreted with caution.

### Effects of candidate variants on HT susceptibility among females 50 years old and above

We note that the genetic effect on BP may be influenced by the level of estrogen [35]. Therefore we reinvestigated the genetic association of the candidate variants among the females aged 50 years old and above. No significant association was observed between these variants with (i) HT (**S9****–****S11 Tables**); (ii) the change of BP, among the older females.

However, both younger and older female carriers of *ADRB2* risk genotype were associated with higher DBP and MAP (**S12** **and** **S13 Tables**). Intriguingly, the *CYP11B2* A-G haplotype between the older and younger female carriers exhibited opposite effects on SBP than other genotypes (P < 0.05). The genetic association of the elevated BP was consistent with the trend shown by the HT males (**S14 Table**). However no appreciative evidence of sex-genotype interactions with all three candidate genes tested.

Collectively, our investigations proposed that genetic variation for *CYP11B2* and *ADRB2* contribute to the elevation of BP in Malay HT, males and females respectively, thence susceptibility of HT, hence may be functionally important.

### Diversity of *CYP11B2* across global populations

We asked if the variability *CYP11B2* variants across different populations (hence population susceptibility to HT) is driven by the need for salt owing to environmental drift, like the *AGT*. If our postulation is true, then these risk variants ideally, would be expected to decline as the geographical latitude coordinate increases [36]; whereas alleles that are in favor of heat preservation should increase as the geographical latitude coordinate increases. To address this, the following correlation analyses were carried out: (i) geographical latitude coordinate vs SBP and BMI; (ii) geographical latitude coordinate vs risk variants; (iii) SBP vs BMI; and (iv) risk variants vs SBP and BMI.

We retrieved the global male BP and BMI variations from the website NCD Risk Factor Collaboration (NCD-RisC; http://ncdrisc.org/index.html).

### Correlation between geographical latitude coordinate with SBP and BMI

We first revealed that both SBP and BMI variations were significantly correlated to geographical latitude (P_SBP_ = 0.04; P_BMI_ = 9.29 x 10^−4^) (**S3** **and** **S4 Figs**; **S15 Table**), suggesting possible latitude attribution with the effectiveness of salt retention and heat preservation.

### Correlation between geographical latitude coordinate with risk variants

The distribution of allele and genotype frequencies for 38 selected populations retrieved from public domains revealed that for both RAAS related genes – *AGT* and *CYP11B2* – the HT risk alleles showed substantial variation between the African and non-African populations, but not for *ADRB2*. Notably, the African and Southeast Asian (SEA) populations where the Malays are located, exhibited similar allele and genotype frequencies for these *AGT* and *CYP11B2* variants (**S16 Table**).

**Fig 2:**
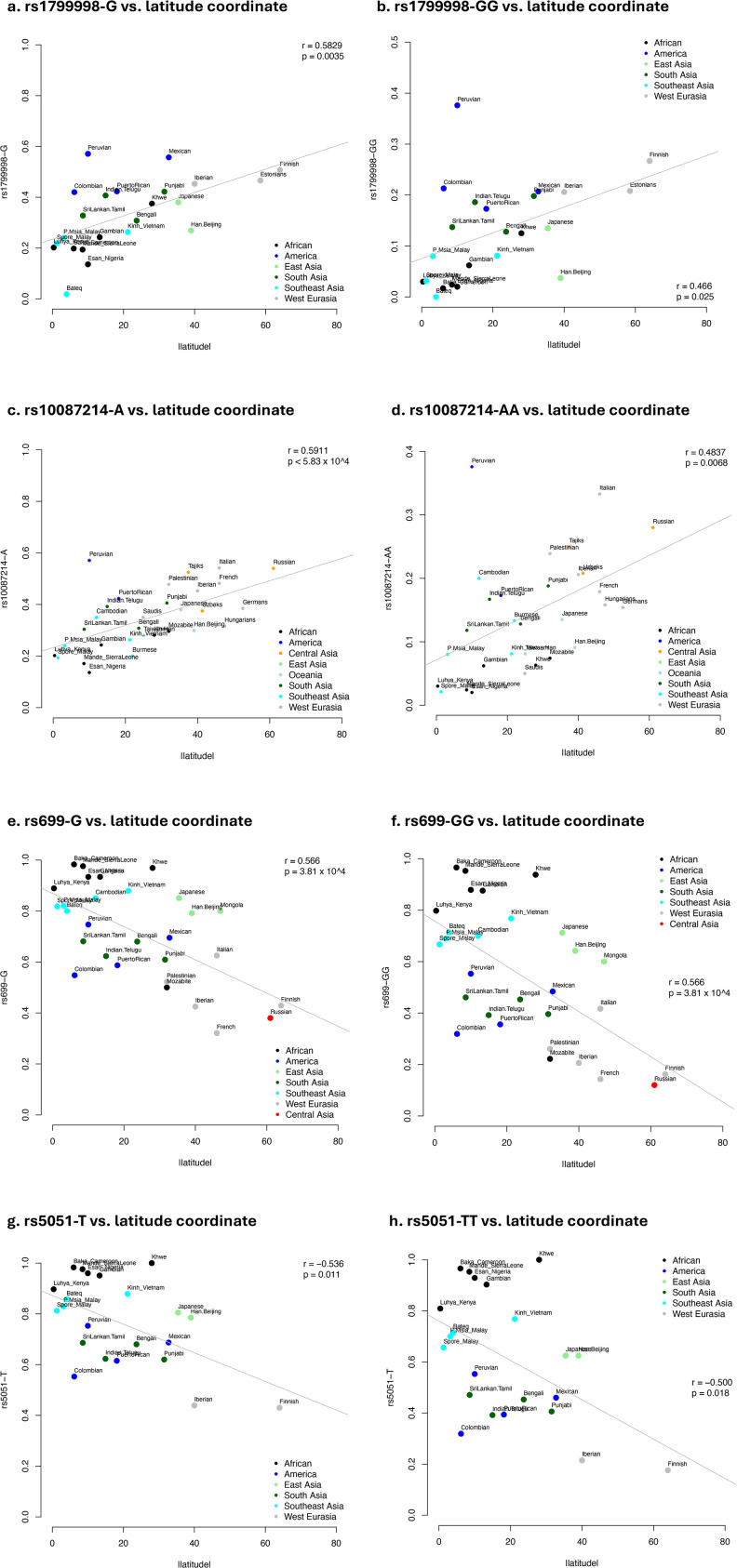
Correlation between the *CYP11B2* ancestral allele frequencies of populations across different continents (Y-axis) and latitude coordinates (X-axis) for the variants. (a) rs1799998 risk allele G; (b) rs1799998 risk genotype GG; (c) rs10087214 risk allele A; (d) rs10087214 risk genotype AA; (e) rs699 risk allele G; (f) rs699 risk genotype GG; (g) rs5051 risk allele T; (h). Allele and genotype frequencies for both SNPs are significantly correlated with the geographical latitude of the studied populations.

The *AGT* risk variants frequencies decreased as the latitude coordinate increased; but *CYP11B2* risk variants were positively correlated with latitude coordinate (**Fig 2; S16 Table**). For *ADRB2*, only rs1042714 was modestly correlated with the distance of the populations from equator (P_allele_ = 0.02; P_genotype_ = 0.04) (**S5 Fig**).

### Correlation between risk variants with SBP and BMI

*AGT* and *ADRB2* (rs1042714 only) were found to be inversely correlated with BMI; whereas *CYP11B2* was positively correlated with BMI distributions of the global populations studied (**S6 Fig**).

### Gene-gene interaction

**Fig 3:**
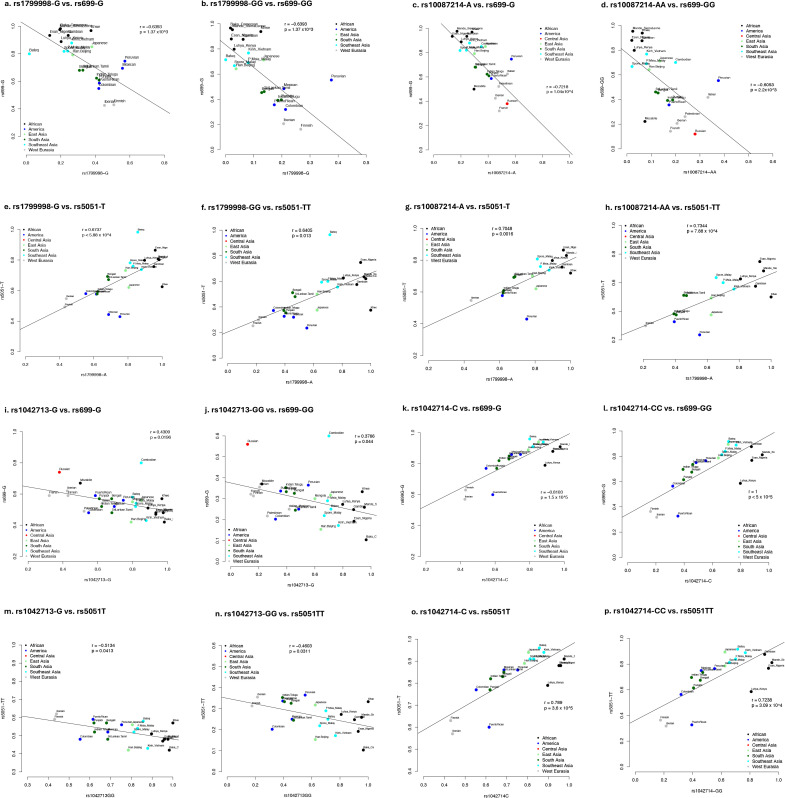
Correlation of the genotype and allele frequencies between *CYP11B2* and *AGT* variants. (a) rs699-G vs. rs1799998-G; (b) rs699-GG vs. rs1799998-GG; (c) rs699-G vs. rs10087214-A; (d) rs699-GG vs. rs10087214-GG.

We hypothesized that if the salt-sensitivity phenotype associated with the RAAS variations co-evolved under the same selection pressures, similar geographical distribution in these unlinked genes would be expected [37]. Both *CYP11B2* variant frequencies were negatively correlated with *AGT* (rs699; P < 0.001); while *ADRB2* (rs1042714 only) was positively correlated with *AGT* (P < 1 x 10^−5^) but negatively correlated with *CYP11B2* (P = 0.0128), consistent with the correlation trend with BMI (**Fig 3**). These collective observations therefore imply plausible interactions between these genes that are under the same selective pressure in response to climatic drift.

### Analysis of Tajima’s D

When the *AGT* and *CYP11B2* genes sequences from the Malay were analyzed, Tajima’s D and Fu and Li’s D* and F* did not deviate significantly from expectations under neutrality (**Table 3**). However, our understanding that the rs699 and rs1799998 contribute to significant phenotypic effects and their strong correlation with the geographical coordinates [6] led us to postulate that natural selection might have operated on these sites.

**Table 3:** Estimates of nucleotide diversity, natural selection and neutrality tests of *AGT* and *CYP11B2.*

Gene	regionID	chr	start	end	Fu & Li’s D*	Fu & Li’s F*	Tajima’s D	Tajima’s D Pvalue	Theta pi	Theta K	Fu & Li’s D* proteinP	Fu & Li’s F* proteinP	Tajima’s D proteinP
*CYP11B2*	ENSG00000179142	8	143,993,117	143,998,116	1.5096	2.5909	2.9789	0.0055	3.4088	1.7027	0.1158	0.0327	0.0689
*AGT*	ENSG00000135744	1	230,853,299	230,858,298	1.7011	3.2565	4.0952	0.0001	2.7327	1.1238	0.0675	0.0057	0.0058

Tajima’s D < 0 (theoretical P < 1 x 10^−3^) indicates an excess of rare variants compared with common variants. The positive Tajima’s D values (Theta pi > Theta K) observed in the Malays suggested lack of rare alleles and plausible balancing selection signal on both the *AGT* and *CYP11B2* variants, as supported by the Fu and Li’s D’ statistics (**Table 3**; **S7 Fig**). The effect of demography was then “normalized” based on the chromosome-wide protein coding genic data (chromosome 1 for *AGT*, and chromosome 8 for *CYP11B2*); and the empirical P value computed remained significant for *AGT* (P = 5 x 10^−3^), however the significance of the *CYP11B2* signal became modest (P = 0.0689) (**Table 3**). An excess of intermediate-frequency alleles relative to what is expected under neutrality was observed (Positive Fu and Li’s F value, P = 0.0327), which may imply putative balancing selection, population substructure or population contraction. Overall, our analysis suggests a non-neutral evolution (plausibly due to balancing selection or population substructure) for *CYP11B2*; and suggesting a signal of balancing selection or population substructure for *AGT*, among the Malays. Judging the relatively recent demographic history of the Malays [38,39], we suggest that population substructure may not the reason.

In sum, our analyses suggested that *CYP11B2* genetic variation is associated with HT Malay males and post-menopausal females, likely via the regulation of RAAS which in turned increased the salt and water retention. Although with modest statistical evidence, the varying susceptibility of *CYP11B2* genetic variation to essential HT may suggest a result of population adaptation to climate drift as reflected by geographical latitude.

## Discussion

This study documented (i) an association between *CYP11B2* genetic variation, and the rise of BP in Malay HT, males and post-menopausal females; while *ADRB2* variant was associated with elevated BP in Malay females; and (ii) the relationship between these genetic variants and the distance of the cohort from the equator using global population database, which subsequently demonstrated that the risk allele and genotype frequencies of the Malays are similar to the populations residing the regions close to equator (e.g., African). In the Malay cohort, there was no association between *AGT* and HT. This was anticipated since nearly all Malays were homozygous or heterozygous for what other studies have identified as being the *AGT* risk alleles in these SNPs associated with elevated BP [40–43]. However, the frequency of the *AGT* risk alleles was higher in populations close to the equator, consistent with earlier studies [16,44]. The *CYP11B2* variations also was strongly positively correlated with geographical latitude, and the frequencies of the risk variants were similar to the populations residing in the regions close to equator. In contrast, the risk alleles frequencies of *ADRB2*, is not known to be associated directly with a volume/sodium homeostatic system, were not consistently greater in populations close to the equator.

The variability of BP across different populations could be explained by the “heat adaptation” and the “sodium retention” hypotheses [16,44,45]. The variants known to regulate sodium homeostasis namely, *AGT*-rs699, *CYP3A*5*3, *GNB3*-C835T and *ACE* insertion/deletion (I/D) polymorphism are some of the classical examples [6,16,37,46]. To these genes this study suggests *CYP11B2* as a putative candidate. In addition, we propose the third complementing hypothesis: whilst modern human migrated out of Africa to the colder climate, the selection pressure turned into ‘heat preservation’ to keep body warm, therefore increased fat distribution and elevated BP. To this end, the risk genotypes (except *ADRB2*-rs1042713) were associated with higher BMI and fat distribution [47,48]. Our argument is that the mechanism of heat acclimation results in increased sweat rate and plasma volume but decreased in sodium excretion in sweat and plasma ALDO levels [49,50]. *CYP11B2* risk variant which confers higher ALDO levels therefore would be unfavored in the tropics. As modern human moved toward colder climate, the adaptation to heat preservation elevates ALDO level [51,52], thence increase in *CYP11B2* risk alleles frequencies. The shift of climate could had conferred different etiologies of HT between different populations. Our hypothesis is in part supported by the Tajima’s D analysis, suggesting a putative balancing selection on *AGT* and notably, *CYP11B2*, occurred among the Malays. The long evolutionary history of RAAS has been well noted [44,53–55]. We postulate that the modest selection signal observed for *CYP11B2* variants using Tajima’s D may be due to a historical balancing selection event that occurred far in the past, such that current statistical approaches lack sufficient power to detect it. The statistics that may provide hints towards long-term balancing selection on RAAS (in this case, both *AGT* and *CYP11B2*) include: (i) elevated positive Tajima’s D value; (ii) significant Fu and Li’s F statistics which suggest excess of intermediate frequencies; and (iii) higher pairwise nucleotide diversity (Theta pi) relative to the segregating sites (Theta K). Collectively, we postulate that *CYP11B2* may have acted as (i) antagonistic pleiotropy, whereby high ALDO may be advantageous in colder climate for heat preservation; yet increase risk of HT; or (ii) spatially varying selection, whereby populations in the arid regions may favor alleles that enhance ALDO production to retain water; whereas populations in humid, salt-rich regions might favor alleles that reduce ALDO to lower HT risk. However, we acknowledge that the current evidence is insufficient to draw any conclusion hence further advanced analyses are required to warrant the conclusion the presence of the balancing selection of RAAS.

We revealed associations between *CYP11B2* variants with increased BP among Malay HT males and older females, reiterated the power of *CYP11B2* haplotype in identifying the HT susceptibility [15]. Essentially, the genetic association with HT was revealed via the identification of protective alleles because they are easier to identify than the older HT susceptible alleles. From population genetic perspective, the protective alleles are believed to have risen to high frequencies more recently and therefore are more tightly linked to nearby markers [36]. A meta-analysis claimed that the rs1799998-C/G allele is associated with a 17% reduced relative risk of HT compared with the T/A allele [56]. However, there are conflicting reports as to whether the T or C allele is more prevalent in HT [40,57].

rs10087214-G/A is a promoter variant in strong LD with the rs1799998-C/T, and the G allele was associated with elevated ALDO level and increased risk of HT [3,57,58]. Nonetheless our results yielded contradictory findings. The effect of rs1799998-G/rs10087214-A haplotype was stronger with a SBP elevation among the males, consistent with several earlier findings [8,59]. The reason for these contradictions is uncertain although numerous hypotheses have been proposed. First, population heterogeneity is a likely cause. Nearly all reported studies were population- not controlled-based. The phenotype of genes involved in sodium/volume homeostasis and secondarily BP levels are likely highly dependent on the quality of the environmental controls used and particularly the criteria used to define a “normal”. On this background, others have reported associations between allele variants of *CYP11B2* and decreased baroreflex sensitivity [60] and contradicting results with ALDO levels in HT [61]. Further investigations with more comprehensive HT phenotypic characterization are warranted to confirm this speculation.

Intriguingly, while the *CYP11B2* variants frequencies are similar between both sexes, their effects on BP alterations differed. The female G-A haplotype carriers showed an opposite effect compared to male carriers, i.e., average lower BP as opposed to those carrying haplotypes other than G-A (**S8 Table**). However, further analysis revealed that older females carrying AA-GG diplotype expressed lower SBP, consistent with the observation in males (**S13 Table**). This is in line with the general understanding that the sex hormones (e.g., estrogen) protect against HT in younger females [31,35], which may partly explain the inconsistency of genetic association between *CYP11B2* and HT.

*In vitro* studies showed that the *CYP11B2* rs1799998-C allele binds to the steroidogenic factor 1 (SF-1) with approximately four times higher affinity than the T allele, possibly altering the transcription rate of *CYP11B2* [58], which plausibly explained the opposite effect of the G-A haplotype between males and females although precise mechanism yet to be elucidated. Further, androgen was reported to have direct effect on RAAS and on sodium reabsorption in the proximal tubule of the nephron [62,63], hence conceivable to speculate that the *CYP11B2* G-A haplotype may have a stronger impact towards the susceptibility of HT among males.

The two *ADRB2* SNPs cause a disproportionate regulation level of ALDO secretion during liberal salt intake, therefore alters the ANG II function, leading to impaired renin response, hence dysregulated salt and water retention [64]. Notably, rs1042713 attenuates vasodilator response to catecholamine and impaired peripheral vascular resistance [11]; while rs1042714-CC reduces vascular reactivity [65]. The fact that we failed to replicate the haplotype effect as reported previously [15], because LD between the two SNPs in the Malays was negligible, as opposed to the Caucasians (r^2^ = 0.4).

To the best of our knowledge, this study is likely the first genetic association study of HT associated genes conducted on a well-characterized Malay population, which revealed the effects of these variations on biological sex. We suggest *CYP11B2* as another potential candidate that underwent forces of natural selection. Furthermore, this study also provides an explanation to the variability of mean BP across all populations globally.

Several study limitations are noted. First, larger sample size for case-control study may be required to confirm the effect on the biological sex on the changes of BP in Malays. Second, owing to the limited clinical data available (plasma ALDO and renin levels, renal plasma flow), the HT intermediate phenotypes such as low renin, non-modulation salt-sensitive, or normal/high renin salt-sensitive, could not be characterized. Although we did not measure SS status or RAAS intermediates in this study, prior evidence provides biological plausibility for a mechanistic role of *CYP11B2* variants in SS-HT [66]. Functional studies further show that transcription factors such as CREB/ATF, SF-1, and NURR1 regulate *CYP11B2* promoter activity in response to Ang II, linking variation at this locus to ALDO biosynthesis and sodium handling [67]. While our findings are consistent with these mechanisms, confirmation in the Malay or neighboring Southeast Asian populations will require direct assessment of renin, ALDO, and salt-sensitivity phenotypes in future studies. Third, we recognize that our analysis is limited only to six SNPs underlying the three SSBP associated genes. Additional SNPs may allow haplotype analysis to be carried out to capture more genetic variations for HT. Fourth, to account for multiple comparisons, we applied Holm–Bonferroni, Storey’s q-value, and permutation-based corrections across all SNPs tested. As anticipated, given the modest sample size and limited number of tests, none of the nominal associations remained statistically significant after adjustment (e.g., *CYP11B2* rs10087214: P = 0.010; FDR-adjusted q ≈ 0.16). Nonetheless, because this was a hypothesis-driven candidate gene study focused on a limited set of biologically plausible variants, the lack of significance after correction likely reflects the conservative nature of such adjustments in small-scale studies rather than an inflated false-positive rate. Therefore, the modest associations, particularly at *CYP11B2,* remain noteworthy and warrant replication in larger, independently sampled Malay populations. Fifth, because our analyses were based on aggregated data from publicly available sources, covariates such as BMI and blood pressure could not be adjusted at the individual level. Therefore, these correlations should be interpreted with caution, as they provide only a general impression rather than definitive evidence. Lastly, we note that the evidence of natural selection of *CYP11B2* may be inconclusive, and further detailed analyses using whole genome sequencing data may be required.

In summary, this study delivers the following messages: differential susceptibility of SS-HT (i) varies across populations of different ancestries correlated with geographical coordinates, plausibly attributed to the drift of climate, resulting in differential heat adaptation and sodium homeostasis on the one hand and temperature and energy storage on another hand, implying that the shift of climate could had conferred different etiologies of HT between different populations; and (ii) attributes to different phenotypic characterizations (sex, hormones) even within a population of single ancestry. We echo that development of pharmacogenetic marker(s) to predict responsiveness of anti-HT medication requires comprehensive characterization of population genetics and HT intermediate (or sub-intermediate) phenotypes.

## Supporting information

S1 Table Alternative names for the SNPs of interest. (DOCX)

S2 Table Haplotype and diplotype association analyses of the AGT, CYP11B2 and ABDR2 genetic variants with HT individuals. G-A haplotype frequency is significantly higher in the normotensive group.
(DOCX)

S3 Table Haplotype and diplotype frequencies of AGT, CYP11B2, and ADRB2 genetic variants among the HT individuals. (DOCX)

S4 Table Demographic and clinical data between HT and NT individuals in all samples (males + females), between sex, younger (less than 50 years old) and older (more than 50 years old) females. (DOCX)

S5 Table Analysis of genetic association between (a) AGT-rs699 and rs5051 with all the HT individuals (males + females), between sex (male and female), younger (less than 50 years old) and older (more than 50 years old) females; (b) CYP11B2-rs1799998 and rs10087214; (c) ADRB2-rs1042713 and rs1042714. Chi-square analysis was performed, and the P value was statistically adjusted with logistic regression (LR) (parentheses) confounding covariates including age, BMI, blood glucose, lipid profiles and history of anti-hypertension medications).
(DOCX)

S6 Table Genotypes for the CYP11B2 variants and the changes of mean systolic blood pressure (SBP), diastolic blood pressure (DBP) and mean arterial pressure (MAP) in HT. (DOCX)

S7 Table Association of genotypes for the AGT, CYP11B2 and ADRB2 variants and the changes of mean systolic blood pressure (SBP), diastolic blood pressure (DBP) and mean arterial pressure (MAP) in HT. Individuals with rs10087214-AA were shown to have average higher BP than other genotypes.
(DOCX)

S8 Table Association of AGT, CYP11B2 and ADRB2 haplotypes and diplotypes of the HT individuals and the changes of mean systolic blood pressure (SBP), diastolic blood pressure (DBP) and mean arterial pressure (MAP) in HT. Male carriers with CYP11B2 G/A haplotype had significantly higher mean SBP, DBP, and MAP. Male carriers with CYP11B2 GG/AA diplotype exhibited significantly higher mean DBP and MAP.
(DOCX)

S9 Table Association analysis of AGT, CYP11B2 and ADRB2 genetic variants with female HT individuals age 50 years old and above. (DOCX)

S10 Table Haplotype and diplotype frequencies distributions of AGT, CYP11B2 and ADRB2 genetic variants for females 50 years old and above. (DOCX)

S11 Table Association of haplotype and diplotype analysis of the AGT, CYP11B2 and ADRB2 genetic variants with females HT individuals age 50 years old and above. (DOCX)

S12 Table Association of genotypes for the AGT, CYP11B2 and ADRB2 variants and the changes of mean systolic blood pressure (SBP), diastolic blood pressure (DBP) and mean arterial pressure (MAP) in HT females age 50 years old and above. (DOCX)

S13 Table Association of AGT, CYP11B2 and ADRB2 variants of the HT females age 50 years and above, and the changes of mean systolic blood pressure (SBP), diastolic blood pressure (DBP) and mean arterial pressure (MAP). Females age 50 years and above that carried AGT rs5051-TT or CYP11B2 rs1799998-AA had average lower SBP as opposed to those who carried other genotypes; whereas carriers of ADRB2 rs1047714-CC had average lower MAP as opposed to those who carried outer genotypes.
(DOCX)

S14 Table Association of CYP11B2 haplotypes and diplotypes of the HT females age 50 years and above, and the changes of mean systolic blood pressure (SBP), diastolic blood pressure (DBP) and mean arterial pressure (MAP). CYP11B2 rs1799998/ rs10087214 AA-GG diplotype had significantly lower SBP as opposed to other diplotypes. AGT rs699/rs5051 G-T haplotype had significantly lower SBP as opposed to other haplotypes, however, the effect of this haplotype is small, by lowering BP measurement only 1 mmHg.
(DOCX)

S15 Table Allele and genotype frequencies of the global populations. Data was predominantly obtained from PGG.SNV database (https://www.pggsnv.org/index.html), 1000 Genomes database, Singapore Genome Variation Project (SGVP) [1], Liu et al (2015) [2] for NGO data, Yew et al (2018) [3] for NB data, and 230 hypertension subjects genotyped with Illumina 660W and OminExpress [4]. ‘-‘, data not available; SBP, systolic blood pressure; DBP, diastolic blood pressure; MAP, mean arterial pressure; BMI, body mass index.
(DOCX)

S16 Table Differentiation of allele and genotype frequencies between African populations and the non-African populations. Significantly different allele and genotype frequencies were observed between African and non-African populations; but not between African and Southeast Asian populations.
(DOCX)

S1 Fig Linkage disequilibrium between the variants for AGT (rs699 and rs5051), CYP11B2 (rs1799998 and rs10087214) and ADRB2 (rs1042713 and rs1042714). Weak r2 was observed between the ADRB2-rs1042713 and rs1042714.
(DOCX)

S2 Fig Effect of CYP11B2 variants to the changes of BP in (a) males; (b) females; (c) all. AA genotype exhibited significantly higher SBP, DBP and MAP among the Malay HT males, but no significant difference between the Malay HT females.
(DOCX)

S3 Fig Correlation between BP and latitude coordinate. The mean BP of the global male populations was retrieved from NCD Risk Factor Collaboration (NCD-RisC; http://ncdrisc.org/index.html). Only the mean BP of males was included for subsequent analysis.
(DOCX)

S4 Fig Correlation between BMI and latitude coordinate. The mean BMI of the global male populations was retrieved from NCD Risk Factor Collaboration (NCD-RisC; http://ncdrisc.org/index.html). We only included the mean BMI of males.
(DOCX)

S5 Fig Frequencies of the risk alleles and genotypes for the SNPs ADRB2- rs1042713 and rs1042714 and their correlation with geographical latitude. (a) rs1042713; (b) r1042714.
(DOCX)

S6 Fig Frequencies of the risk alleles and genotypes of AGT, CYP11B2 and ADRB2, and their correlation with body mass index. (a) AGT-rs699; (b) CYP11B2-rs1799998; (c) CYP11B2-rs10087214; (d) ADRB2-rs1042713; (e) ADRB2-rs1042714.
(DOCX)

S7 Fig Tajima’s D for AGT and CYP11B2 among the Malays. Y axis indicates -log_10_(theoretical P values); X axis indicates the positions of the sliding windows. Each single panel corresponds to sliding window size.
(DOCX)
