# Supplementary material for: Genetic Association of the Renin-Angiotensin-Aldosterone System with hypertension among the Malays and their adaptation to climate change
Source: PLoS One. 2026 Apr 15;21(4):e0346614. doi: 10.1371/journal.pone.0346614 (PMC13082722; doi:10.1371/journal.pone.0346614)
Supplement: S1 Table — (DOCX) [file pone.0346614.s001.docx]

**S1 Table. Alternative names for the SNPs of interest.**

| **Gene** | **rsID#** | **Alternative names** |
| --- | --- | --- |
| *AGT* | rs699 | M235T |
|  | rs5051 | A-6C |
| *CYP11B2* | rs1799998 | C-344T |
|  | rs10087214 | G-470A |
| *ADRB2* | rs1042713 | G16R; G46A |
|  | rs1042714 | Q27E; C79G |

Owing to the strong linkage on the SNPs of interest, the following text shall denote the risk alleles for AGT rs699-G, rs5051-T as ‘AGT genotype’ or ‘AGT allele’; and CYP11B2 rs179999-G and rs10087214-A as ‘CYP11B2 genotype’ or ‘CYP11B2 allele’, unless otherwise stated.
