# Supplementary material for: Genetic Association of the Renin-Angiotensin-Aldosterone System with hypertension among the Malays and their adaptation to climate change
Source: PLoS One. 2026 Apr 15;21(4):e0346614. doi: 10.1371/journal.pone.0346614 (PMC13082722; doi:10.1371/journal.pone.0346614)
Supplement: S2 Table — G-A haplotype frequency is significantly higher in the normotensive group. (DOCX) [file pone.0346614.s002.docx]

**S2 Table. Haplotype and diplotype association analyses of the *AGT*, *CYP11B2* and *ABDR2* genetic variants with HT individuals.** G-A haplotype frequency is significantly higher in the normotensive group.

| **Gene** | **rsID#** |  | **Frequencies** | | | | | | | | |
| --- | --- | --- | --- | --- | --- | --- | --- | --- | --- | --- | --- |
|  |  |  | **Female** | | | **Male** | | | **All** | | |
|  |  |  | **HTN** | **NT** | **p-value** | **HTN** | **NT** | **p-value** | **HTN** | **NT** | **p-value**  **(LR)** |
| ***AGT*** | **rs699/**  **rs5051** | **Haplotype** | **(N = 484)** | **(N = 472)** | **(LR)** | **(N = 464)** | **(N = 358)** | **(LR)** | **(N = 948)** | **(N = 830)** |  |
|  |  | G-T | 0.69 (336) | 0.71 (334) | 0.672 | 0.69 (321) | 0.68 (241) | 0.597 | 0.71 (657) | 0.70 (575) | 0.99 |
|  |  | Others | 0.31 (148) | 0.29 (138) | (0.631) | 0.31 (143) | 0.32 (117) | (0.204) | 0.29 (291) | 0.30 (255) | (0.229) |
|  |  | **Diplotype** | **(N = 242)** | **(N = 236)** |  | **(N = 232)** | **(N = 179)** |  | **(N = 427)** | **(N = 415)** |  |
|  |  | GG-TT | 0.69 (165) | 0.70 (163) | 0.844 | 0.67 (154) | 0.66 (118) | 1.000 | 0.65 (319) | 0.67 (281) | 0.943 |
|  |  | Others | 0.32 (77) | 0.30 (73) | (0.749) | 0.33 (78) | 0.34 (61) | (0.553) | 0.35 (155) | 0.33 (134) | (0.614) |
| ***CYP11B2*** | **rs1799998/**  **rs10087214** | **Haplotype** | **(N = 474)** | **(N = 474)** |  | **(N = 380)** | **(N = 356)** |  | **(N = 854)** | **(N = 830)** |  |
|  |  | G-A | 0.06 (27) | 0.08 (39) | 0.16 | 0.08 (31) | 0.08 (30) | 1.000 | 0.07 (60) | 0.08 (73) | 0.181 |
|  |  | Others | 0.94 (447) | 0.92 (435) | (0.179) | 0.92 (349) | 0.92 (326) | (0.221) | 0.93 (749) | 0.92 (757) | (0.147) |
|  |  | **Diplotype** | **(N = 237)** | **(N = 237)** |  | **(N = 190)** | **(N = 178)** |  | **(N = 427)** | **(N = 415)** |  |
|  |  | GG-AA | 0.05 (13) | 0.07 (18) | 0.711 | 0.07 (15) | 0.07 (14) | 1.000 | 0.07 (27) | 0.08 (32) | 0.5 |
|  |  | Others | 0.95 (224) | 0.93 (219) | (0.551) | 0.93 (175) | 0.93 (163) | (0.471) | 0.93 (400) | 0.92 (383) | (0.212) |
| ***ADRB2*** | **rs1042713/**  **rs1042714** | **Haplotype** | **(N = 484)** | **(N = 476)** |  | **(N = 430)** | **(N = 476)** |  | **(N = 914)** | **(N = 832)** |  |
|  |  | G-C | 0.48 (233) | 0.48 (227) | 0.897 | 0.47 (203) | 0.46 (165) | 0.830 | 0.47 (432) | 0.53 (390) | 0.871 |
|  |  | Others | 0.52 (251) | 0.52 (249) | (0.865) | 0.53 (227) | 0l54 (190 | (0.511) | 0.53 (482) | 0.47 (832) | (0.948) |
|  |  | **Diplotype** | **(N = 242)** | **(N = 238)** |  | **(N = 190)** | **(N = 178)** |  | **(N = 457)** | **(N = 416)** |  |
|  |  | GG-CC | 0.19 (46) | 0.21 (50) | 0.648 | 0.17 (38) | 0.16 (27) | 0.801 | 0.18 (84) | 0.19 (77) | 1.000 |
|  |  | Others | 0.81 (196) | 0.79 (188) | (0.331) | 0.83 (117) | 0.84 (151) | (0.511) | 0.82 (373) | 0.71 (339) | (0.568) |

HTN, hypertension; NT, normotension.
