# Supplementary material for: Genetic Association of the Renin-Angiotensin-Aldosterone System with hypertension among the Malays and their adaptation to climate change
Source: PLoS One. 2026 Apr 15;21(4):e0346614. doi: 10.1371/journal.pone.0346614 (PMC13082722; doi:10.1371/journal.pone.0346614)
Supplement: S3 Table — (DOCX) [file pone.0346614.s003.docx]

**S3 Table. Haplotype and diplotype frequencies of *AGT*, *CYP11B2*, and *ADRB2* genetic variants among the HT individuals.**

| **Gene** | **rsID#** |  | **Frequency** | | | | | |
| --- | --- | --- | --- | --- | --- | --- | --- | --- |
|  |  |  | **All** | | **Male** | | **Female** | |
|  |  |  | **HT** | **NT** | **HT** | **NT** | **HT** | **NT** |
| ***AGT*** | **rs699/ rs5051** | **Haplotype** | **(N = 948)** | **(N = 830)** | **(N = 464)** | **(N = 358)** | **(N = 484)** | **(N = 472)** |
|  |  | A/C | 0.01 (7) | 0.03 (32) | 0.02 (8) | 0.04 (16) | 0.02 (9) | 0.03 (16) |
|  |  | G/C | 0.13 (131) | 0.13 (108) | 0.14 (64) | 0.13 (48) | 0.14 (67) | 0.13 (60) |
|  |  | A/T | 0.15 (143) | 0.14 (115) | 0.15 (71) | 0.15 (53) | 0.15 (72) | 0.13 (62) |
|  |  | G/T | 0.71 (657) | 0.70 (575) | 0.69 (321) | 0.68 (241) | 0.69 (336) | 0.71 (334) |
|  |  | **Diplotype** | **(N = 474)** | **(N = 415)** | **(N = 232)** | **(N = 179)** | **(N = 242)** | **(N = 236)** |
|  |  | AA/CC | 0.02 (8) | 0.04 (16) | 0.02 (4) | 0.04 (8) | 0.02 (4) | 0.03 (8) |
|  |  | AA/TC | 0.01 (1) | - | - | - | 0.005 (1) | - |
|  |  | AA/TT | 0.01 (1) | 0.01 (1) | - | 0.01 (1) | 0.005 (1) | - |
|  |  | AG/TC | 0.26 (126) | 0.25 (104) | 0.26 (61) | 0.26 (47) | 0.02 (65) | 0.24 (57) |
|  |  | AG/TT | 0.03 (14) | 0.02 (9) | 0.04 (10) | 0.02 (4) | 0.02 (4) | 0.02 (5) |
|  |  | GG/TC | 0.02 (5) | 0.01 (4) | 0.01 (3) | 0.01 (1) | 0.02 (2) | 0.01 (3) |
|  |  | GG/TT | 0.65 (319) | 0.67 (281) | 0.67 (154) | 0.66 (118) | 0.68 (165) | 0.70 (163) |
| ***CYP11B2*** | **rs1799998/ rs10087214** | **Haplotype** | **(N = 854)** | **(N = 830)** | **(N = 380)** | **(N = 356)** | **(N = 474)** | **(N = 474)** |
|  |  | A-A | 0.19 (164) | 0.14 (119) | 0.20 (75) | 0.14 (50) | 0.19 (89) | 0.15 (70) |
|  |  | A-G | 0.53 (454) | 0.61 (503) | 0.52 (169) | 0.62 (220) | 0.55 (259) | 0.60 (285) |
|  |  | G-A | 0.07 (60) | 0.08 (73) | 0.08 (31) | 0.08 (30) | 0.06 (27) | 0.08 (39) |
|  |  | G-G | 0.21 (176) | 0.17 (135) | 0.20 (78) | 0.16 (56) | 0.20 (99) | 0.17 (80) |
|  |  | **Diplotype** | **(N = 427)** | **(N = 415)** | **(N = 190)** | **(N = 178)** | **(N = 237)** | **(N = 237)** |
|  |  | AA-AA | 0.01 (1) | - | - | - | 0.01 (1) | - |
|  |  | AA-GA | 0.02 (6) | - | 0.04 (6) | - | - | - |
|  |  | AA-GG | 0.50 (216) | 0.60 (247) | 0.47 (91) | 0.61 (108) | 0.53 (125) | 0.58 (139) |
|  |  | GA-GA | 0.36 (157) | 0.28 (120) | 0.36 (70) | 0.28 (50) | 0.36 (87) | 0.30 (70) |
|  |  | GA-GG | 0.03 (16) | 0.03 (11) | 0.04 (7) | 0.02 (4) | 0.03 (9) | 0.03 (7) |
|  |  | GG-AA | 0.07 (27) | 0.07 (32) | 0.07 (15) | 0.07 (14) | 0.05 (13) | 0.07 (18) |
|  |  | GG-GA | 0.01 (4) | 0.02 (5) | 0.02 (1) | 0.02 (2) | 0.02 (3) | 0.02 (3) |
| ***ARDRB2*** | **rs1042713/ rs1042714** | **Haplotype** | **(N = 914)** | **(N = 832)** | **(N = 430)** | **(N = 356)** | **(N = 242)** | **(N = 238)** |
|  |  | A/G | 0.04 (33) | 0.03 (26) | 0.04 (19) | 0.04 (15) | 0.03 (14) | 0.02 (11) |
|  |  | G/G | 0.05 (44) | 0.06 (49) | 0.04 (16) | 0.05 (16) | 0.05 (26) | 0.07 (32) |
|  |  | A/C | 0.44 (405) | 0.44 (367) | 0.45 (192) | 0.45 (160) | 0.44 (211) | 0.43 (206) |
|  |  | G/C | 0.47 (432) | 0.47 (390) | 0.47 (203) | 0.46 (165) | 0.48 (233) | 0.48 (227) |
|  |  | **Diplotype** | **(N = 457)** | **(N = 416)** | **(N = 215)** | **(N = 178)** | **(N = 242)** | **(N = 238)** |
|  |  | AA/CC | 0.22 (104) | 0.23 (96) | 0.21 (47) | 0.22 (39) | 0.23 (57) | 0.24 (57) |
|  |  | GA/CC | 0.42 (196) | 0.42 (175) | 0.45 (98) | 0.46 (82) | 0.40 (98) | 0.39 (93) |
|  |  | GA/CG | 0.08 (31) | 0.06 (26) | 0.08 (19) | 0.08 (15) | 0.05 (14) | 0.04 (11) |
|  |  | GA/GG | 0.01 (2) | - | - | - | - | - |
|  |  | GA/CC | 0.18 (84) | 0.19 (77) | 0.17 (38) | 0.15 (27) | 0.19 (46) | 0.21 (50) |
|  |  | GG/CG | 0.08 (38) | 0.08 (36) | 0.06 (11) | 0.07 (14) | 0.13 (27) | 0.09 (22) |
|  |  | GG/GG | 0.01 (2) | 0.02 (6) | 0.03 (2) | 0.02 (1) | - | 0.03 (5) |

HT, hypertensive; NT, normotensive.
