# Supplementary material for: Genetic Association of the Renin-Angiotensin-Aldosterone System with hypertension among the Malays and their adaptation to climate change
Source: PLoS One. 2026 Apr 15;21(4):e0346614. doi: 10.1371/journal.pone.0346614 (PMC13082722; doi:10.1371/journal.pone.0346614)
Supplement: S4 Table — (DOCX) [file pone.0346614.s004.docx]

**S4 Table. Demographic and clinical data between HT and NT individuals in all samples (males + females), between sex, younger (less than 50 years old) and older (more than 50 years old) females.**

|  | **All** | | | **Male** | | | **Female** | | | **Younger Female** | | | **Older Female** | | | |
| --- | --- | --- | --- | --- | --- | --- | --- | --- | --- | --- | --- | --- | --- | --- | --- | --- |
| **Parameters** | **HT**  **(N = 313)** | **NT**  **(N = 418)** | **p-value** | **HT**  **(N = 180)** | **NT**  **(N = 179)** | **p-value** | **HT**  **(N = 133)** | **NT**  **(N = 239)** | **p-value** | **HT**  **(N = 55)** | **NT**  **(N = 144)** | **p-value** | **HT**  **(N = 78)** | **NT**  **(N = 95)** | **P-value** |  |
| **Age (years)** | 53.6 ± 8.7 | 49.7 ± 8.0 | <0.001* | 54.8 ± 8.4 | 51.0 ± 8.0 | <0.001* | 51.9 ± 8.7 | 48.7 ± 7.8 | <0.001* | 43.4 ± 4.0 | 43.5 ± 3.8 | 0.879 | 58.0 ± 5.5 | 56.5 ± 5.4 | 0.091 |  |
| **BMI (kg/m^2^)** | 25.3 ± 2.7 | 24.0 ± 3.2 | <0.001* | 25.2 ± 2.5 | 23.8 ± 3.0 | <0.001* | 25.4 ± 3.0 | 24.1 ± 3.3 | <0.001* | 25.3 ± 3.1 | 24.0 ± 3.1 | 0.007* | 25.4 ± 2.9 | 24.3 ± 3.5 | 0.0037* |  |
| **SBP (mmHg)** | 153.3 ± 15.3 | 115.8 ± 7.5 | <0.001* | 154.2 ± 15.5 | 116.5 ± 7.7 | <0.001* | 152.0 ± 15.0 | 115.4 ± 7.4 | <0.001* | 148.5 ± 17.8 | 114.3 ± 7.5 | <0.001* | 154.4 ± 12.2 | 117.0 ± 6.9 | <0.001* |  |
| **DBP (mmHg)** | 89.1 ± 10.4 | 71.8 ± 6.5 | <0.001* | 89.6 ± 8.8 | 71.5 ± 6.3 | <0.001* | 88.5 ± 11.0 | 72.1 ± 6.6 | <0.001* | 93.5 ± 9.5 | 72.6 ± 6.5 | <0.001* | 85.0 ± 10.6 | 71.3 ± 6.6 | <0.001* |  |
| **MAP (mmHg)** | 110.5 ± 9.8 | 86.5 ± 6.0 | <0.001* | 111.1 ± 9.4 | 86.5 ± 6.0 | <0.001* | 109.7 ± 10.2 | 86.5 ± 6.0 | <0.001* | 111.8 ± 11.3 | 86.5 ± 6.1 | <0.001* | 108.1 ± 9.1 | 86.5 ± 5.9 | <0.001* |  |
| **Waist circumferences (cm)** | 85.3 ± 10.1 | 79.9 ± 11.6 | <0.001* | 88.2 ± 9.9 | 83.6 ± 10.7 | <0.001* | 81.3 ± 9.1 | 77.0 ± 11.4 | <0.001* | 80.2 ± 10.1 | 75.0 ± 12.1 | 0.005* | 82.1 ± 8.3 | 80.2 ± 9.6 | 0.165 |  |
| **Glucose (mmol/l)** | 4.9 ± 1.0 | 4.8 ± 0.6 | 0.570 | 5.0 ± 0.9 | 4.9 ± 0.7 | 0.348 | 4.7 ± 1.2 | 4.8 ± 0.6 | 0.452 | 4.5 ± 1.4 | 4.6 ± 0.6 | 0.716 | 4.8 ± 1.0 | 4.9 ± 0.5 | 0.165 |  |
| **High density lipid (HDL) (mg/dL)** | 1.1 ± 0.4 | 1.3 ± 0.3 | <0.001* | 1.0 ± 0.3 | 1.1 ± 0.2 | 0.157 | 1.3 ± 0.4 | 1.4 ± 0.3 | 0.008* | 1.2 ± 0.5 | 1.4 ± 0.3 | 0.006* | 1.3 ± 0.4 | 1.3 ± 0.3 | 0.430 |  |
| **Low density lipid (LDL) (mg/dL)** | 3.7 ± 1.2 | 3.8 ± 0.9 | <0.001* | 3.7 ± 1.2 | 3.9 ± 0.9 | 0.164 | 3.2 ± 1.3 | 3.7 ± 0.9 | 0.254 | 3.3 ± 1.3 | 3.6 ± 0.8 | 0.102 | 3.7 ± 1.2 | 3.8 ± 1.1 | 0.498 |  |
| **Total cholesterol (TC) (mg/dL)** | 5.6 ± 1.5 | 5.8 ± 1.0 | <0.001* | 5.7 ± 1.5 | 5.8 ± 1.1 | 0.552 | 5.5 ± 1.6 | 5.7 ± 1.0 | 0.119 | 5.3 ± 1.6 | 5.6 ± 0.9 | 0.155 | 5.6 ± 1.6 | 5.9 ± 1.2 | 0.166 |  |
| **Triglyceride (TG) (mg/dL)** | 1.7 ± 1.0 | 1.4 ± 0.7 | 0.002* | 1.9 ± 1.0 | 1.6 ± 0.7 | 0.008* | 1.4 ± 0.8 | 1.3 ± 0.7 | 0.248 | 1.3 ± 0.6 | 1.1 ± 0.5 | 0.048* | 1.5 ± 0.9 | 1.6 ± 0.9 | 0.171 |  |
| **Sex:**  **Male**  **Female** | 180  133 | 179  239 | <0.001* | - | - | - | - | - | - | **-** | **-** | **-** | **-** | **-** | **-** |  |
