# Supplementary material for: Genetic Association of the Renin-Angiotensin-Aldosterone System with hypertension among the Malays and their adaptation to climate change
Source: PLoS One. 2026 Apr 15;21(4):e0346614. doi: 10.1371/journal.pone.0346614 (PMC13082722; doi:10.1371/journal.pone.0346614)
Supplement: S5 Table — Chi-square analysis was performed, and the P value was statistically adjusted with logistic regression (LR) (parentheses) confounding covariates including age, BMI, blood glucose, lipid profiles and history of anti-hypertension medications). (DOCX) [file pone.0346614.s005.docx]

**S5 Table. Analysis of genetic association between (a) *AGT*-rs699 and rs5051 with all the HT individuals (males + females), between sex (male and female), younger (less than 50 years old) and older (more than 50 years old) females; (b) *CYP11B2*-rs1799998 and rs10087214; (c) *ADRB2*-rs1042713 and rs1042714.** Chi-square analysis was performed, and the P value was statistically adjusted with logistic regression (LR) (parentheses) confounding covariates including age, BMI, blood glucose, lipid profiles and history of anti-hypertension medications).

(a).

| **Gene** | **rsID#** |  | **All** | | | **Male** | | | **Female** | | | **Younger Female** | | | **Older Female** | | |
| --- | --- | --- | --- | --- | --- | --- | --- | --- | --- | --- | --- | --- | --- | --- | --- | --- | --- |
|  |  |  | **HT** | **NT** | **p-value**  **(LR)** | **HT** | **NT** | **p-value**  **(LR)** | **HT** | **NT** | **p-value**  **(LR)** | **HT** | **NT** | **p-value**  **(LR)** | **HT** | **NT** | **p-value**  **(LR)** |
| ***AGT*** | rs699 (M235T) | **Genotype** | **N = 312** | **N = 415** |  | **N = 179** | **N = 179** |  | **N = 133** | **N = 236** |  | **N = 55** | **N = 144** |  | **N = 78** | **N = 92** |  |
|  |  | AA | 0.02 (9) | 0.04 (17) | 0.542 | 0.2 (4) | 0.05 (9) | 0.339 | 0.03 (5) | 0.03 (8) | 0.727 | 0.05 (3) | 0.02 (3) | 0.431 | 0.03 (2) | 0.05 (5) | 0.437 |
|  |  | AG | 0.25 (79) | 0.27 (113) | (0.230) | 0.27 (49) | 0.29 (51) | (1.000) | 0.23 (30) | 0.26 (62) | (0.985) | 0.24 (13) | 0.27 (39) | (1.000) | 0.22 (17) | 0.25 (23) | (1.000) |
|  |  | GG | 0.71 (224) | 0.69 (285) |  | 0.71 (126) | 0.66 (119) |  | 0.74 (98) | 0.71 (166) |  | 0.71 (39) | 0.71 (102) |  | 0.75 (59) | 0.70 (64) |  |
|  |  | **Allele** | **N = 624** | **N = 830** |  | **N = 358** | **N = 358** |  | **N = 266** | **N = 472** |  | **N = 120** | **N = 288** |  | **N = 146** | **N = 184** |  |
|  |  | A | 0.15 (96) | 0.17 (142) | 0.379 | 0.16 (57) | 0.18 (64) | 0.485 | 0.15 (39) | 0.17 (78) | 0.506 | 0.17 (20) | 0.18 (45) | 0.793 | 0.13 (19) | 0.17 (33) | 0.223 |
|  |  | G* | 0.85 (528) | 0.83 (688) | (0.490) | 0.84 (301) | 0.82 (294) | (0.367) | 0.85 (227) | 0.83 (394) | (0.209) | 0.83 (100) | 0.82 (243) | (0.701) | 0.87 (127) | 0.83 (151) | (0.156) |
|  | rs5051 (A-6G) | **Genotype** | **N = 288** | **N = 417** |  | **N = 159** | **N = 179** |  | **N = 129** | **N = 238** |  | **N = 55** | **N = 144** |  | **N = 74** | **N = 94** |  |
|  |  | TT | 0.73 (210) | 0.70 (293) | 0.387 | 0.71 (113) | 0.69 (123) | 0.263 | 0.75 (97) | 0.71 (170) | 0.698 | 0.71 (39) | 0.72 (103) | 0.820 | 0.78 (58) | 0.71 (67) | 0.318 |
|  |  | TC | 0.25 (72) | 0.26 (108) | (0.484) | 0.27 (43) | 0.26 (48) | (1.000) | 0.23 (29) | 0.25 (60) | (0.985) | 0.26 (14) | 0.26 (38) | (1.000) | 0.21 (15) | 0.23 (22) | (1.000) |
|  |  | CC | 0.02 (6) | 0.04 (16) |  | 0.02 (3) | 0.05 (8) |  | 0.02 (3) | 0.04 (8) |  | 0.03 (2) | 0.02 (3) |  | 0.01 (1) | 0.06 (5) |  |
|  |  | **Allele** | **N = 584** | **N = 826** |  | **N = 318** | **N = 350** |  | **N = 266** | **N = 476** |  | **N = 120** | **N = 288** |  | **N = 146** | **N = 188** |  |
|  |  | T* | 0.85 (498) | 0.84 (691) | 0.410 | 0.85 (269) | 0.83 (292) | 0.682 | 0.86 (229) | 0.84 (399) | 0.412 | 0.83 (100) | 0.84 (243) | 0.793 | 0.88 (129) | 0.83 (156) | 0.168 |
|  |  | C | 0.15 (86) | 0.16 (135) | (0.418) | 0.15 (49) | 0.17 (58) | (0.534) | 0.14 (37) | 0.16 (77) | (0.303) | 0.17 (20) | 0.16 (45) | (0.734) | 0.12 (17) | 0.17 (32) | (0.192) |

(b)

| **Gene** | **rsID#** |  | **All** | | | **Male** | | | **Female** | | | **Younger Female** | | | **Older Female** | | |
| --- | --- | --- | --- | --- | --- | --- | --- | --- | --- | --- | --- | --- | --- | --- | --- | --- | --- |
|  |  |  | **HT** | **NT** | **p-value**  **(LR)** | **HT** | **NT** | **p-value**  **(LR)** | **HT** | **NT** | **p-value**  **(LR)** | **HT** | **NT** | **p-value**  **(LR)** | **HT** | **NT** | **p-value**  **(LR)** |
| ***CYP11B2*** | rs1799998 (-344G/A) | **Genotype** | **N = 249** | **N = 416** |  | **N = 124** | **N = 178** |  | **N = 125** | **N = 238** |  | **N = 54** | **N = 143** |  | **N = 71** | **N = 95** |  |
|  |  | GG | 0.08 (19) | 0.08 (37) | **0.042*** | 0.08 (10) | 0.09 (16) | 0.151 | 0.07 (9) | 0.09 (21) | 0.246 | 0.13 (7) | 0.08 (12) | 0.311 | 0.03 (2) | 0.10 (9) | 0.181 |
|  |  | GA | 0.41 (103) | 0.32 (132) | (0.327) | 0.41 (51) | 0.30 (54) | (1.000) | 0.42 (52) | 0.33 (78) | (0.731) | 0.37 (20) | 0.31 (43) | (1.000) | 0.45 (32) | 0.37 (35) | (1.000) |
|  |  | AA | 0.51 (127) | 0.60 (96) |  | 0.51 (63) | 0.61 (108) |  | 0.51 (64) | 0.58 (139) |  | 0.50 (27) | 0.59 (88) |  | 0.52 (37) | 0.53 (51) |  |
|  |  | **Allele** | **N = 512** | **N = 832** |  | **N = 248** | **N = 340** |  | **N = 264** | **N = 476** |  | **N = 120** | **N = 286** |  | **N = 144** | **N = 190** |  |
|  |  | G* | 0.25 (130) | 0.25 (205) | 0.953 | 0.71 (177) | 0.26 (87) | 0.411 | 0.22 (59) | 0.25 (119) | 0.419 | 0.23 (27) | 0.23 (67) | 0.840 | 0.22 (32) | 0.27 (52) | 0.283 |
|  |  | A | 0.75 (382) | 0.75 (627) | (0.558) | 0.29 (71) | 0.74 (253) | (0.900) | 0.78 (205) | 0.75 (357) | (0.416) | 0.77 (93) | 0.77 (219) | (0.836) | 0.78 (112) | 0.73 (137) | (0.546) |
|  | rs10087214  (-470G/A) | **Genotype** | **N = 311** | **N = 415** |  | **N = 178** | **N = 178** |  | **N = 133** | **N = 237** |  | **N = 55** | **N = 143** |  | **N = 78** | **N = 94** |  |
|  |  | GG | 0.54 (169) | 0.62 (258) | **0.010*** | 0.55 (98) | 0.63 (112) | 0.119 | 0.53 (71) | 0.62 (146) | 0.069 | 0.53 (29) | 0.64 (91) | 0.342 | 0.54 (42) | 0.59 (55) | 0.141 |
|  |  | GA | 0.41 (126) | 0.30 (125) | (0.221) | 0.39 (70) | 0.29 (52) | (1.000) | 0.42 (56) | 0.31 (73) | (0.731) | 0.38 (21) | 0.28 (40) | (1.000) | 0.45 (35) | 0.35 (33) | (1.000) |
|  |  | AA | 0.05 (16) | 0.08 (32) |  | 0.06 (10) | 0.08 (14) |  | 0.05 (6) | 0.07 (18) |  | 0.09 (5) | 0.08 (12) |  | 0.01 (1) | 0.06 (6) |  |
|  |  | **Allele** | **N = 622** | **N = 830** |  | **N = 356** | **N = 358** |  | **N = 264** | **N = 474** |  | **N = 120** | **N = 286** |  | **N = 144** | **N = 188** |  |
|  |  | G | 0.77 (476) | 0.77 (640) | 0.799 | 0.75 (266) | 0.75 (270) | 0.785 | 0.79 (210) | 0.77 (364) | 0.389 | 0.81 (97) | 0.78 (222) | 0.472 | 0.78 (113) | 0.76 (142) | 0.529 |
|  |  | A* | 0.23 (144) | 0.23 (190) | (0.463) | 0.25 (90) | 0.25 (88) | (0.915) | 0.21 (54) | 0.23 (110) | (0.593) | 0.19 (23) | 0.22 (64) | (0.553) | 0.22 (31) | 0.24 (46) | (0.990) |

**(c)**

| **Gene** | **rsID#** |  | **All** | | | **Male** | | | **Female** | | | **Younger Female** | | | **Older Female** | | |
| --- | --- | --- | --- | --- | --- | --- | --- | --- | --- | --- | --- | --- | --- | --- | --- | --- | --- |
|  |  |  | **HT** | **NT** | **p-value**  **(LR)** | **HT** | **NT** | **p-value**  **(LR)** | **HT** | **NT** | **p-value**  **(LR)** | **HT** | **NT** | **p-value**  **(LR)** | **HT** | **NT** | **p-value**  **(LR)** |
| ***ADRB2*** | rs1042713 (G16R) | **Genotype** | **N = 311** | **N = 416** |  | **N = 178** | **N = 178** |  | **N = 133** | **N = 238** |  | **N = 55** | **N = 143** |  | **N = 78** | **N = 95** |  |
|  |  | GG | 0.29 (90) | 0.29 (119) | 0.775 | 0.27 (48) | 0.24 (42) | 0.513 | 0.31 (42) | 0.32 (77) | 0.942 | 0.27 (15) | 0.33 (47) | 0.627 | 0.35 (27) | 0.32 (30) | 0.874 |
|  |  | GA | 0.50 (156) | 0.48 (201) | (0.314) | 0.56 (99) | 0.55 (97) | (1.000) | 0.43 (57) | 0.44 (104) | (0.699) | 0.46 (25) | 0.45 (65) | (1.000) | 0.41 (32) | 0.41 (39) | (1.000) |
|  |  | AA | 0.21 (65) | 0.23 (96) |  | 0.17 (31) | 0.21 (39) |  | 0.26 (34) | 0.24 (57) |  | 0.27 (15) | 0.22 (31) |  | 0.24 (19) | 0.27 (26) |  |
|  |  | **Allele** | **N = 620** | **N = 832** |  | **N = 356** | **N = 358** |  | **N = 264** | **N = 476** |  | **N = 120** | **N = 286** |  | **N = 144** | **N = 190** |  |
|  |  | G* | 0.53 (331) | 0.53 (439) | 0.955 | 0.55 (195) | 0.52 (186) | 0.450 | 0.52 (136) | 0.54 (258) | 0.483 | 0.48 (58) | 0.55 (159) | 0.181 | 0.54 (78) | 0.52 (99) | 0.709 |
|  |  | A | 0.47 (289) | 0.47 (393) | (0.653) | 0.45 (161) | 0.48 (172) | (0.647) | 0.48 (128) | 0.46 (218) | (0.806) | 0.52 (62) | 0.45 (127) | (0.726) | 0.46 (66) | 0.48 (91) | (0.500) |
|  | rs1042714  (Q27E) | **Genotype** | **N = 280** | **N = 416** |  | **N = 150** | **N = 178** |  | **N = 130** | **N = 238** |  | **N = 54** | **N = 143** |  | **N = 76** | **N = 95** |  |
|  |  | CC | 0.84 (236) | 0.84 (348) | 0.677 | 0.85 (127) | 0.83 (148) | 0.659 | 0.84 (109) | 0.84 (200) | 0.220 | 0.87 (47) | 0.81 (116) | 0.382 | 0.82 (84) | 0.88 (84) | 0.234 |
|  |  | CG | 0.15 (42) | 0.15 (62) | (0.416) | 0.14 (21) | 0.16 (29) | (1.000) | 0.16 (21) | 0.14 (33) | (0.858) | 0.13 (7) | 0.16 (23) | (1.000) | 0.18 (14) | 0.11 (10) | (1.000) |
|  |  | GG | 0.10 (2) | 0.01 (6) |  | 0.01 (2) | 0.01 (1) |  | 0.00 (0) | 0.02 (5) |  | 0.00 (0) | 0.03 (4) |  | 0.00 (0) | 0.01 (1) |  |
|  |  | **Allele** | **N = 564** | **N = 832** |  | **N = 300** | **N = 350** |  | **N = 264** | **N = 476** |  | **N = 120** | **N = 286** |  | **N = 144** | **N = 190** |  |
|  |  | C* | 0.92 (519) | 0.91 (758) | 0.627 | 0.92 (275) | 0.92 (321) | 0.983 | 0.92 (244) | 0.91 (433) | 0.496 | 0.90 (108) | 0.91 (255) | 0.802 | 0.94 (136) | 0.94 (178) | 0.772 |
|  |  | G | 0.08 (45) | 0.09 (74) | (0.654) | 0.08 (25) | 0.08 (29) | (0.759) | 0.08 (20) | 0.09 (43) | (0.898) | 0.10 (12) | 0.09 (31) | (0.796) | 0.06 (8) | 0.06 (12) | (0.472) |
