# Supplementary material for: Genetic Association of the Renin-Angiotensin-Aldosterone System with hypertension among the Malays and their adaptation to climate change
Source: PLoS One. 2026 Apr 15;21(4):e0346614. doi: 10.1371/journal.pone.0346614 (PMC13082722; doi:10.1371/journal.pone.0346614)
Supplement: S6 Table — (DOCX) [file pone.0346614.s006.docx]

**S6 Table. Genotypes for the *CYP11B2* variants and the changes of mean systolic blood pressure (SBP), diastolic blood pressure (DBP) and mean arterial pressure (MAP) in HT.**

| **Gene** | **rsID#** | **M/F/A** | **Genotype** | **N** | **BP** | | |
| --- | --- | --- | --- | --- | --- | --- | --- |
|  |  |  |  |  | **SBP**  **(Mean/ SD)** | **DBP**  **(Mean/SD)** | **MAP (Mean/SD)** |
| ***AGT*** | **rs699** | **M** | **AA** | 4 | 152.3 ± 16.4 | 87.1 ± 19.1 | 108.9 ± 18.0 |
|  |  |  | **AG** | 49 | 154.8 ± 14.1 | 88.4 ± 8.6 | 110.5 ± 8.5 |
|  |  |  | **GG** | 126 | 154.0 ± 16.2 | 90.2 ± 8.5 | 111.5 ± 9.5 |
|  |  | **F** | **AA** | 5 | 148.7 ± 8.1 | 84.1 ± 8.5 | 105.6 ± 6.0 |
|  |  |  | **AG** | 30 | 152.7 ± 16.1 | 89.1 ± 12.1 | 110.3 ± 10.6 |
|  |  |  | **GG** | 98 | 151.9 ± 15.0 | 88.5 ± 10.7 | 109.7 ± 10.2 |
|  |  | **A** | **AA** | 9 | 150.3 ± 11.7 | 85.4 ± 13.3 | 107.0 ± 11.9 |
|  |  |  | **AG** | 79 | 154.0 ± 14.8 | 88.7 ± 10.0 | 110.4 ± 9.3 |
|  |  |  | **GG** | 224 | 153.1 ± 15.7 | 89.5 ± 9.5 | 110.7 ± 9.9 |
|  | **rs5051** | **M** | **TT** | 113 | 154.6 ± 16.7 | 89.9 ± 8.9 | 111.4 ± 9.9 |
|  |  |  | **TC** | 43 | 153.8 ± 13.0 | 88.8 ± 8.4 | 110.5 ± 7.9 |
|  |  |  | **CC** | 3 | 153.5 ± 20.0 | 85.1 ± 23.0 | 107.9 ± 22.0 |
|  |  | **F** | **TT** | 97 | 151.4 ± 14.8 | 88.5 ± 10.8 | 109.5 ± 10.2 |
|  |  |  | **TC** | 29 | 153.7 ± 16.2 | 88.8 ± 12.9 | 110.5 ± 11.1 |
|  |  |  | **CC** | 3 | 150.8 ± 10.6 | 87.3 ± 3.5 | 108.4 ± 1.7 |
|  |  | **A** | **TT** | 210 | 153.1 ± 15.9 | 89.3 ± 9.8 | 110.5 ± 10.1 |
|  |  |  | **TC** | 72 | 153.8 ± 14.3 | 88.8 ± 10.4 | 110.5 ± 9.2 |
|  |  |  | **CC** | 6 | 152.1 ± 14.4 | 86.2 ± 14.7 | 108.2 ± 13.9 |
| ***CYP11B2*** | **rs1799998** | **M** | **GG** | 10 | 16 1.8 ± 17.4 | 94.0 ± 12.7 | 116.5 ± 13.2 |
|  |  |  | **GA** | 51 | 152.5 ± 15.3 | 87.6 ± 8.8 | 109.3 ± 8.7 |
|  |  |  | **AA** | 63 | 153.7 ± 15.8 | 90.2 ± 8.4 | 111.4 ± 9.7 |
|  |  | **F** | **GG** | 9 | 144.2 ± 6.1 | 86.0 ± 9.9 | 105.4 ± 5.4 |
|  |  |  | **GA** | 52 | 152.6 ± 15.7 | 88.5 ± 11.2 | 109.9 ± 9.7 |
|  |  |  | **AA** | 64 | 152.9 ± 15.4 | 89.1 ± 11.5 | 110.4 ± 11.4 |
|  |  | **A** | **GG** | 19 | 153.4 ± 15.8 | 90.2 ± 11.9 | 111.3 ± 11.5 |
|  |  |  | **GA** | 103 | 152.6 ± 15.4 | 88.1 ± 10.1 | 109.6 ± 9.2 |
|  |  |  | **AA** | 127 | 153.3 ± 15.6 | 89.7 ± 10.1 | 110.9 ± 10.3 |
|  | **rs10087214** | **M** | **GG** | 98 | 155.0 ± 16.1 | 90.4 ± 8.3 | 111.2 ± 9.4 |
|  |  |  | **GA** | 70 | 151.9 ± 14.3 | 87.8 ± 8.7 | 109.2 ± 8.5 |
|  |  |  | **AA** | 10 | 163.8 ± 17.1 | 95.8 ± 10.9 | 118.4 ± 11.9 |
|  |  | **F** | **GG** | 71 | 152.6 ± 15.0 | 88.8 ± 11.3 | 110.1 ± 11.1 |
|  |  |  | **GA** | 56 | 152.0 ± 15.6 | 88.3 ± 10.7 | 109.5 ± 9.4 |
|  |  |  | **AA** | 6 | 143.7 ± 4.0 | 86.1 ± 9.4 | 105.3 ± 5.9 |
|  |  | **A** | **GG** | 169 | 154.0 ± 15.6 | 89.8 ± 9.7 | 111.2 ± 10.1 |
|  |  |  | **GA** | 126 | 152.0 ± 14.8 | 88.0 ± 9.6 | 109.3 ± 8.9 |
|  |  |  | **AA** | 16 | 156.2 ± 16.7 | 92.2 ± 11.1 | 113.5 ± 11.8 |
| ***ADRB2*** | **rs1042713** | **M** | **GG** | 48 | 155.3 ± 16.4 | 89.2 ± 9.9 | 111.2 ± 10.5 |
|  |  |  | **GA** | 99 | 153.7 ± 15.5 | 89.6 ± 8.9 | 111.0 ± 9.6 |
|  |  |  | **AA** | 31 | 154.3 ± 14.9 | 90.8 ±6.4 | 112.0 ± 6.8 |
|  |  | **F** | **GG** | 42 | 155.6 ± 18.4 | 89.8 ± 14.5 | 111.7 ± 13.6 |
|  |  |  | **GA** | 57 | 151.4 ± 11.9 | 88.6 ± 9.0 | 109.5 ± 8.3 |
|  |  |  | **AA** | 34 | 148.4 ± 14.4 | 86.8 ± 8.6 | 107.3 ± 7.7 |
|  |  | **A** | **GG** | 90 | 155.4 ± 17.3 | 89.5 ± 12.2 | 111.5 ± 12.0 |
|  |  |  | **GA** | 156 | 152.9 ± 14.3 | 89.2 ± 9.0 | 110.4 ± 9.1 |
|  |  |  | **AA** | 65 | 151.2 ± 14.9 | 88.7 ± 7.8 | 109.6 ± 7.6 |
|  | **rs1042714** | **M** | **CC** | 127 | 153.7 ± 15.8 | 89.0 ± 8.5 | 110.6 ± 9.3 |
|  |  |  | **CG** | 21 | 157.4 ± 17.2 | 92.3 ± 9.6 | 114.0 ± 10.8 |
|  |  |  | **GG** | 2 | 152.0 ± 18.3 | 97.6 ± 10.8 | 115.7 ± 13.3 |
|  |  | **F** | **CC** | 109 | 150.9 ± 14.9 | 87.4 ± 10.8 | 108.6 ± 9.9 |
|  |  |  | **CG** | 21 | 159.0 ± 14.1 | 94.4 ± 10.7 | 115.9 ± 10.4 |
|  |  |  | **GG** | - | - | - | - |
|  |  | **A** | **CC** | 236 | 152.4 ± 15.4 | 88.3 ± 9.7 | 109.6 ± 9.6 |
|  |  |  | **CG** | 42 | 158.2 ± 15.6 | 93.3 ± 10.1 | 114.9 ± 10.5 |
|  |  |  | **GG** | 2 | 152.0 ± 18.3 | 97.6 ± 10.8 | 115.7 ± 13.3 |

M, male; F, female; A, all; SBP, systolic blood pressure; DBP, diastolic blood pressure; MAP, mean arterial pressure.
