# Supplementary material for: Genetic Association of the Renin-Angiotensin-Aldosterone System with hypertension among the Malays and their adaptation to climate change
Source: PLoS One. 2026 Apr 15;21(4):e0346614. doi: 10.1371/journal.pone.0346614 (PMC13082722; doi:10.1371/journal.pone.0346614)
Supplement: S7 Table — Individuals with rs10087214-AA were shown to have average higher BP than other genotypes. (DOCX) [file pone.0346614.s007.docx]

**S7 Table.** **Association of genotypes for the *AGT*, *CYP11B2* and *ADRB2* variants and the changes of mean systolic blood pressure (SBP), diastolic blood pressure (DBP) and mean arterial pressure (MAP) in HT.** Individuals with rs10087214-AA were shown to have average higher BP than other genotypes.

| **Gene** | **rsID#** | **M/F/A** | **N** | **Genotype** | **SBP** | **p-value (SBP)** | **DBP (Mean/SD)** | **p-value (DBP)** | **MAP (Mean/SD)** | **p-value (MAP)** |
| --- | --- | --- | --- | --- | --- | --- | --- | --- | --- | --- |
|  |  |  |  |  | **(Mean/ SD)** |  |  |  |  |  |
| ***AGT*** | **rs699** | M | 53 | GG | 154.6 ± 14.2 | 0.826 | 90.2 ± 8.5 | 0.197 | 111.5 ± 9.5 | 0.497 |
|  |  |  | 126 | AA + AG | 154.0 ± 15.7 |  | 88.3 ± 9.5 |  | 110.4 ± 9.2 |  |
|  |  | F | 98 | GG | 151.9 ± 15.0 | 0.941 | 88.5 ± 10.7 | 0.927 | 109.7 ± 10.2 | 0.979 |
|  |  |  | 35 | AA + AG | 152.1 ± 15.2 |  | 88.3 ± 11.7 |  | 109.6 ± 10.2 |  |
|  |  | A | 224 | GG | 153.1 ± 15.7 | 0.792 | 89.5 ± 9.5 | 0.356 | 110.7 ± 9.9 | 0.636 |
|  |  |  | 88 | AA + AG | 153.6 ± 14.5 |  | 88.3 ± 10.4 |  | 110.1 ± 9.6 |  |
|  | **rs5051** | M | 113 | TT | 154.6 ± 16.7 | 0.764 | 89.9 ± 8.9 | 0.427 | 111.4 ± 9.9 | 0.511 |
|  |  |  | 46 | TC + CC | 153.8 ± 13.2 |  | 88.6 ± 9.5 |  | 110.3 ± 8.9 |  |
|  |  | F | 97 | TT | 151.4 ± 14.8 | 0.499 | 88.5 ± 10.8 | 0.946 | 109.5 ± 10.2 | 0.705 |
|  |  |  | 32 | TC + CC | 153.5 ± 15.7 |  | 88.7 ± 12.3 |  | 110.3 ± 10.6 |  |
|  |  | A | 210 | TT | 153.1 ± 15.9 | 0.797 | 89.3 ± 9.8 | 0.645 | 110.5 ± 10.1 | 0.862 |
|  |  |  | 78 | TC + CC | 153.6 ± 14.2 |  | 88.6 ± 10.7 |  | 110.3 ± 9.6 |  |
| ***CYP11B2*** | **rs1799998** | M | 10 | GG | 161.8 ± 17.4 | 0.102 | 94.0 ± 12.7 | 0.105 | 116.5 ± 13.2 | 0.059 |
|  |  |  | 114 | GA + AA | 153.2 ± 15.5 |  | 89.1 ± 8.6 |  | 110.4 ± 9.3 |  |
|  |  | F | 9 | GG | 144.2 ± 6.1 | 0.102 | 86.0 ± 9.9 | 0.473 | 105.4 ± 5.4 | 0.19 |
|  |  |  | 116 | GA + AA | 152.8 ± 15.5 |  | 88.8 ± 11.4 |  | 110.1 ± 10.7 |  |
|  |  | A | 19 | GG | 153.4 ± 15.8 | 0.906 | 90.2 ± 11.9 | 0.614 | 111.2 ± 11.2 | 0.703 |
|  |  |  | 230 | GA + AA | 153.0 ± 15.5 |  | 89.0 ± 10.1 |  | 110.3 ± 10.0 |  |
|  | **rs10087214** | M | 10 | AA | 163.8 ± 17.1 | ***0.049**** | 95.8 ± 10.9 | ***0.024**** | 118.4 ± 11.9 | ***0.013**** |
|  |  |  | 168 | GG + GA | 153.7 ± 15.4 |  | 89.3 ± 8.5 |  | 110.8 ± 9.1 |  |
|  |  | F | 6 | AA | 143.7 ± 4.0 | 0.169 | 86.1 ± 9.4 | 0.59 | 105.3 ± 5.9 | 0.288 |
|  |  |  | 127 | GG + GA | 152.4 ± 15.2 |  | 88.6 ± 11.0 |  | 109.9 ± 10.3 |  |
|  |  | A | 16 | AA | 156.2 ± 16.7 | 0.436 | 92.2 ± 11.8 | 0.213 | 113.5 ± 11.8 | 0.217 |
|  |  |  | 295 | GG + GA | 153.1 ± 15.3 |  | 89.0 ± 9.7 |  | 110.4 ± 9.6 |  |
| ***ADRB2*** | **rs1042713** | M | 48 | GG | 155.3 ± 16.4 | 0.595 | 89.2 ± 9.9 | 0.658 | 111.2 ± 10.5 | 0.989 |
|  |  |  | 130 | GA + AA | 153.9 ± 15.3 |  | 89.9 ± 8.4 |  | 111.2 ± 9.0 |  |
|  |  | F | 42 | GG | 155.6 ± 18.4 | 0.056 | 89.8 ± 14.6 | 0.452 | 111.7 ± 13.6 | 0.19 |
|  |  |  | 91 | GA + AA | 150.3 ± 12.9 |  | 87.9 ± 8.9 |  | 108.7 ± 8.1 |  |
|  |  | A | 90 | GG | 155.4 ± 17.3 | 0.113 | 89.5 ± 12.2 | 0.779 | 111.5 ± 12.0 | 0.298 |
|  |  |  | 221 | GA + AA | 152.4 ± 14.4 |  | 89.1 ± 8.6 |  | 110.2 ± 8.7 |  |
|  | **rs1042714** | M | 127 | CC | 153.7 ± 15.8 | 0.382 | 89.0 ± 8.5 | 0.058 | 110.6 ± 9.3 | 0.099 |
|  |  |  | 23 | CG + GG | 156.9 ± 17.0 |  | 92.8 ± 9.5 |  | 114.1 ± 10.7 |  |
|  |  | F | 109 | CC | 150.9 ± 14.9 | ***0.024**** | 87.4 ± 10.8 | ***0.008**** | 108.6 ± 9.9 | ***0.003**** |
|  |  |  | 21 | CG + GG | 159.0 ± 14.1 |  | 94.4 ± 10.7 |  | 115.9 ± 10.4 |  |
|  |  | A | 236 | CC | 152.4 ± 15.4 | ***0.032**** | 88.3 ± 9.7 | ***0.001**** | 109.6 ± 9.6 | ***0.001**** |
|  |  |  | 44 | CG + GG | 157.9 ± 15.5 |  | 93.5 ± 10.0 |  | 115.1 ± 10.4 |  |

M, male; F, female; A, all; SBP, systolic blood pressure; DBP, diastolic blood pressure; MAP, mean arterial pressure.
