# Supplementary material for: Genetic Association of the Renin-Angiotensin-Aldosterone System with hypertension among the Malays and their adaptation to climate change
Source: PLoS One. 2026 Apr 15;21(4):e0346614. doi: 10.1371/journal.pone.0346614 (PMC13082722; doi:10.1371/journal.pone.0346614)
Supplement: S8 Table — Male carriers with CYP11B2 G/A haplotype had significantly higher mean SBP, DBP, and MAP. Male carriers with CYP11B2 GG/AA diplotype exhibited significantly higher mean DBP and MAP. (DOCX) [file pone.0346614.s008.docx]

**S8 Table. Association of *AGT, CYP11B2 and ADRB2* haplotypes and diplotypes of the HTN individuals and the changes of mean systolic blood pressure (SBP), diastolic blood pressure (DBP) and mean arterial pressure (MAP) in HT.** Male carriers with *CYP11B2* G/A haplotype had significantly higher mean SBP, DBP, and MAP. Male carriers with *CYP11B2* GG/AA diplotype exhibited significantly higher mean DBP and MAP.

| **Gene** | **rsID#** |  | **M/F/A** | **N** | **SBP** | **p-value** | **DBP** | **p-value (DBP)** | **MAP** | **p-value** |
| --- | --- | --- | --- | --- | --- | --- | --- | --- | --- | --- |
|  |  |  |  |  | **(Mean/ SD)** | **(SBP)** | **(Mean/SD)** |  | **(Mean/SD)** | **(MAP)** |
| ***AGT*** | **rs699/ rs5051** | **Haplotype** |  |  |  |  |  |  |  |  |
|  |  | G-T | M | 221 | 154.3 ± 16.6 | 0.973 | 89.9 ± 8.8 | 0.193 | 111.4 ± 9.9 | 0.428 |
|  |  | Others |  | 97 | 154.4 ± 13.7 |  | 88.5 ± 9.5 |  | 110.5 ± 9.1 |  |
|  |  | G-T | F | 191 | 151.6 ± 14.9 | 0.588 | 88.6 ± 10.8 | 0.994 | 109.6 ± 10.3 | 0.786 |
|  |  | Others |  | 67 | 152.8 ± 15.3 |  | 88.6 ± 11.9 |  | 110.0 ± 10.3 |  |
|  |  | G-T | A | 412 | 153.1 ± 15.9 | 0.643 | 89.3 ± 9.8 | 0.406 | 110.6 ± 10.1 | 0.753 |
|  |  | Others |  | 164 | 153.7 ± 14.4 |  | 88.5 ± 10.5 |  | 110.3 ± 9.6 |  |
|  |  | **Diplotype** |  |  |  |  |  |  |  |  |
|  |  | GG-TT | M | 105 | 154.2 ± 16.6 | 0.838 | 90.1 ± 8.9 | 0.268 | 111.4 ± 9.9 | 0.562 |
|  |  | Others |  | 54 | 154.7 ± 14.2 |  | 88.4 ± 9.3 |  | 110.5 ± 9.1 |  |
|  |  | GG-TT | F | 93 | 151.5 ± 15.0 | 0.674 | 88.6 ± 11.0 | 0.947 | 109.6 ± 10.4 | 0.873 |
|  |  | Others |  | 36 | 152.8 ± 15.1 |  | 88.5 ± 16.5 |  | 109.9 ± 10.0 |  |
|  |  | GG-TT | A | 198 | 152.9 ± 15.9 | 0.609 | 89.4 ± 9.9 | 0.448 | 110.6 ± 10.2 | 0.809 |
|  |  | Others |  | 90 | 154.0 ± 14.5 |  | 88.4 ± 10.2 |  | 110.3 ± 9.4 |  |
| ***CYP11B2*** | **rs1799998/ rs10087214** | **Haplotype** |  |  |  |  |  |  |  |  |
|  |  | G-A | M | 19 | 162.4 ± 17.2 | ***0.015**** | 94.9 ± 11.9 | ***0.047**** | 117.4 ± 12.6 | ***0.029**** |
|  |  | Others |  | 229 | 153.2 ± 15.4 |  | 89.0 ± 8.9 |  | 110.4 ± 9.3 |  |
|  |  | G-A | F | 15 | 144.0 ± 5.2 | ***<0.001**** | 86.1 ± 9.4 | 0.363 | 105.4 ± 5.4 | 0.089 |
|  |  | Others |  | 235 | 152.7 ± 15.4 |  | 88.8 ± 11.3 |  | 110.1 ± 10.6 |  |
|  |  | G-A | A | 34 | 154.3 ± 16.1 | 0.63 | 91.0 ± 11.6 | 0.247 | 112.1 ± 11.7 | 0.307 |
|  |  | Others |  | 464 | 152.9 ± 15.4 |  | 88.9 ± 10.1 |  | 110.2 ± 10.0 |  |
|  |  | **Diplotype** |  |  |  |  |  |  |  |  |
|  |  | GG-AA | M | 9 | 163.1 ± 17.9 | 0.071 | 96.0 ± 11.6 | ***0.025**** | 118.4 ± 12.7 | ***0.018**** |
|  |  | Others |  | 115 | 153.2 ± 15.4 |  | 89.0 ± 8.7 |  | 110.4 ± 9.3 |  |
|  |  | GG-AA | F | 6 | 143.7 ± 4.0 | 0.163 | 86.1 ± 9.4 | 0.58 | 105.3 ± 5.9 | 0.282 |
|  |  | Others |  | 119 | 152.6 ± 15.4 |  | 88.8 ± 11.4 |  | 110.0 ± 10.6 |  |
|  |  | GG-AA | A | 15 | 155.3 ± 16.9 | 0.555 | 92.1 ± 11.5 | 0.242 | 113.1 ± 12.2 | 0.247 |
|  |  | Others |  | 234 | 152.9 ± 15.4 |  | 88.9 ± 10.1 |  | 110.2 ± 9.9 |  |
| ***ADRB2*** | **rs1042713/ rs1042714** | **Haplotype** |  |  |  |  |  |  |  |  |
|  |  | G-C | M | 144 | 154.3 ± 16.6 | 0.896 | 88.9 ± 9.4 | 0.183 | 110.7 ± 10.4 | 0.456 |
|  |  | Others |  | 154 | 154.0 ± 15.4 |  | 90.3 ± 8.1 |  | 111.5 ± 8.8 |  |
|  |  | G-C | F | 125 | 153.2 ± 16.0 | 0.283 | 88.4 ± 12.4 | 0.815 | 110.0 ± 11.5 | 0.721 |
|  |  | Others |  | 135 | 151.2 ± 14.0 |  | 88.7 ± 9.6 |  | 109.5 ± 9.0 |  |
|  |  | G-C | A | 269 | 153.9 ± 16.3 | 0.363 | 88.7 ± 10.9 | 0.285 | 110.4 ± 10.9 | 0.808 |
|  |  | Others |  | 289 | 152.7 ± 14.8 |  | 89.6 ± 8.8 |  | 110.6 ± 8.9 |  |
|  |  | **Diplotype** |  |  |  |  |  |  |  |  |
|  |  | GG-CC | M | 25 | 155.0 ± 18.7 | 0.783 | 87.4 ± 10.6 | 0.178 | 109.9 ± 11.6 | 0.502 |
|  |  | Others |  | 124 | 154.0 ± 15.5 |  | 90.0 ± 8.4 |  | 111.4 ± 9.1 |  |
|  |  | GG-CC | F | 27 | 153.3 ± 19.4 | 0.662 | 85.8 ± 14.9 | 0.152 | 108.3 ± 13.9 | 0.418 |
|  |  | Others |  | 103 | 151.9 ± 13.8 |  | 89.3 ± 9.8 |  | 110.1 ± 9.2 |  |
|  |  | GG-CC | A | 52 | 154.1 ± 18.9 | 0.656 | 86.6 ± 13.0 | 0.108 | 109.1 ± 12.8 | 0.265 |
|  |  | Others |  | 227 | 153.0 ± 14.7 |  | 89.7 ± 9.0 |  | 110.8 ± 9.1 |  |

M, male; F, female; A, all; SBP, systolic blood pressure; DBP, diastolic blood pressure; MAP, mean arterial pressure.
