# Supplementary material for: Genetic Association of the Renin-Angiotensin-Aldosterone System with hypertension among the Malays and their adaptation to climate change
Source: PLoS One. 2026 Apr 15;21(4):e0346614. doi: 10.1371/journal.pone.0346614 (PMC13082722; doi:10.1371/journal.pone.0346614)
Supplement: S9 Table — (DOCX) [file pone.0346614.s009.docx]

**S9 Table. Association analysis of *AGT, CYP11B2* and *ADRB2* genetic variants with female HT individuals age 50 years old and above.** Statistical adjustment was applied using logistics regression (by adjusting the confounding covariates including age, sex, BMI and history of anti-hypertension medication)

| **Gene** | **rsID#** |  | **HT** | **NT** | **p-value**  **(LR)** |
| --- | --- | --- | --- | --- | --- |
| ***AGT*** | rs699 | **Genotype** | **(N = 168)** | **(N = 92)** |  |
|  |  | AA | 0.02 (3) | 0.05 (5) | 0.212 |
|  |  | AG | 0.30 (50) | 0.25 (23) | (1.000) |
|  |  | GG | 0.68 (115) | 0.70 (64) |  |
|  |  | **Allele** | **(N = 336)** | **(N = 184)** |  |
|  |  | A | 0.17 (56) | 0.17 (33) | 0.805 |
|  |  | G | 0.83 (280) | 0.83 (151) | (0.807) |
|  | rs5051 | **Genotype** | **(N = 164)** | **(N = 94)** |  |
|  |  | TT | 0.70 (115) | 0.71 (67) | 0.136 |
|  |  | TC | 0.29 (47) | 0.23 (22) | (1.000) |
|  |  | CC | 0.01 (2) | 0.06 (5) |  |
|  |  | **Allele** | (N = 328) | (N = 188) |  |
|  |  | T | 0.84 (272) | 0.83 (156) | 0.900 |
|  |  | CC | 0.16 (51) | 0.17 (32) | (0.774) |
| ***CYP11B2*** | rs1799998 | **Genotype** | **(N = 160)** | **(N = 95)** |  |
|  |  | GG | 0.05 (8) | 0.10 (9) | 0.33 |
|  |  | GA | 0.42 (67) | 0.35 (32) | (1.000) |
|  |  | AA | 0.53 (85) | 0.55 (51) |  |
|  |  | **Allele** | **(N = 320)** | **(N = 190)** |  |
|  |  | G | 0.27 (83) | 0.23 (53) | 0.332 |
|  |  | A | 0.73 (237) | 0.77 (137) | (0.704) |
|  | rs10087214 | **Genotype** | **(N = 168)** | **(N = 94)** |  |
|  |  | GG | 0.55 (93) | 0.59 (55) | 0.662 |
|  |  | GA | 0.40 (67) | 0.35 (33) | (1.000) |
|  |  | AA | 0.05 (8) | 0.06 (6) |  |
|  |  | **Allele** | **(N = 336)** | **(N = 188)** |  |
|  |  | G | 0.75 (253) | 0.78 (143) | 0.485 |
|  |  | A | 0.25 (83) | 0.22 (45) | (0.9) |
| ***ADRB2*** | rs1042713 | **Genotype** | **(N = 168)** | **(N = 95)** |  |
|  |  | GG | 0.31 (52) | 0.32 (30) | 0.683 |
|  |  | GA | 0.46 (77) | 0.41 (39) | (1.000) |
|  |  | AA | 0.23 (39) | 0.27 (26) |  |
|  |  | **Allele** | **(N = 336)** | **(N = 190)** |  |
|  |  | G | 0.54 (181) | 0.52 (99) | 0.947 |
|  |  | A | 0.46 (155) | 0.48 (91) | (0.953) |
|  | rs1042714 | **Genotype** | **(N = 165)** | **(N = 95)** |  |
|  |  | CC | 0.82 (135) | 0.88 (84) | 0.073 |
|  |  | CG | 0.18 (30) | 0.11 (10) | (1.000) |
|  |  | GG | 0.00 (0) | 0.01 (1) |  |
|  |  | **Allele** | **(N = 330)** | **(N = 190)** |  |
|  |  | C | 0.94 (300) | 0.94 (178) | 0.452 |
|  |  | G | 0.06 (30) | 0.06 (12) | (0.444) |

HT, hypertensive; NT, normotensive.
