# Supplementary material for: Genetic Association of the Renin-Angiotensin-Aldosterone System with hypertension among the Malays and their adaptation to climate change
Source: PLoS One. 2026 Apr 15;21(4):e0346614. doi: 10.1371/journal.pone.0346614 (PMC13082722; doi:10.1371/journal.pone.0346614)
Supplement: S10 Table — (DOCX) [file pone.0346614.s010.docx]

**S10 Table. Haplotype and diplotype frequencies distribution of *AGT, CYP11B2* and *ADRB2* genetic variants for females 50 years old and above.**

| **Gene** | **rsID#** |  | **Freq** | |
| --- | --- | --- | --- | --- |
|  |  |  | **HT** | **NT** |
| ***AGT*** | **rs699/ rs5051** | **Haplotype** | **(N = 328)** | **(N = 184)** |
|  |  | A/C | 0.02 (5) | 0.05 (10) |
|  |  | G/C | 0.14 (46) | 0.12 (22) |
|  |  | A/T | 0.15 (50) | 0.13 (23) |
|  |  | G/T | 0.69 (227) | 0.70 (129) |
|  |  | **Diplotype** | **(N = 164)** | **(N = 184)** |
|  |  | AA/CC | 0.01 (2) | 0.05 (5) |
|  |  | AA/TC | 0.005 (1) | - |
|  |  | AA/TT | - | - |
|  |  | AG/TC | 0.28 (45) | 0.24 (21) |
|  |  | AG/TT | 0.02 (4) | 0.02 (2) |
|  |  | GG/TC | 0.005 (1) | 0.01 (1) |
|  |  | GG/TT | 0.68 (111) | 0.68 (63) |
| ***CYP11B2*** | **rs1799998/ rs10087214** | **Haplotype** | **(N = 320)** | **(N = 188)** |
|  |  | A-A | 0.20 (63) | 0.16 (30) |
|  |  | A-G | 0.54 (174) | 0.56 (106) |
|  |  | G-A | 0.05 (15) | 0.08 (15) |
|  |  | G-G | 0.21 (68) | 0.20 (37) |
|  |  | **Diplotype** | **(N = 160)** | **(N = 94)** |
|  |  | AA/AA | 0.01 (1) | - |
|  |  | AA/GG | 0.53 (84) | 0.54 (51) |
|  |  | GA/GA | 0.38 (61) | 0.32 (30) |
|  |  | GA/GG | 0.03 (6) | 0.04 (4) |
|  |  | GG/AA | 0.04 (7) | 0.06 (6) |
|  |  | GG/GA | 0.01 (1) | 0.04 (3) |
| ***ADRB2*** | **rs1042713/ rs1042714** | **Haplotype** | **(N = 330)** | **(N = 190)** |
|  |  | A/G | 0.03 (12) | 0.02 (3) |
|  |  | G/G | 0.05 (18) | 0.05 (9) |
|  |  | A/C | 0.42 (139) | 0.46 (88) |
|  |  | G/C | 0.50 (161) | 0.47 (90) |
|  |  | **Diplotype** | **(N = 165)** | **(N = 95)** |
|  |  | AA/CC | 0.22 (37) | 0.27 (26) |
|  |  | GA/CC | 0.38 (65) | 0.38 (36) |
|  |  | GA/CG | 0.07 (12) | 0.03 (3) |
|  |  | GA/GG | - | - |
|  |  | GG/CC | 0.20 (33) | 0.23 (22) |
|  |  | GG/CG | 0.13 (18) | 0.07 (7) |
|  |  | GG/GG | - | 0.02 (1) |

HT, hypertensive; NT, normotensive.
