# Supplementary material for: Genetic Association of the Renin-Angiotensin-Aldosterone System with hypertension among the Malays and their adaptation to climate change
Source: PLoS One. 2026 Apr 15;21(4):e0346614. doi: 10.1371/journal.pone.0346614 (PMC13082722; doi:10.1371/journal.pone.0346614)
Supplement: S11 Table — (DOCX) [file pone.0346614.s011.docx]

**S11 Table. Association of haplotype and diplotype analysis of the *AGT, CYP11B2* and *ADRB2* genetic variants with females HT individuals age 50 years old and above.** Statistical adjustment was applied using logistics regression (by adjusting the confounding covariates including age, sex, BMI and history of anti-hypertension medication)

| **Gene** | **rsID#** |  | **HT** | **NT** | **p-value (LR)** |
| --- | --- | --- | --- | --- | --- |
| ***AGT*** | **rs699/ rs5051** | **Haplotype** | **(N = 328)** | **(N = 184)** |  |
|  |  | **G-T** | 0.69 (227) | 0.70 (129) | 0.842 |
|  |  | **Others** | 0.31 (56) | 0.30 (55) | (0.986) |
|  |  | **Diplotype** | **(N = 164)** | **(N = 92)** |  |
|  |  | **GG-TT** | 0.68 (111) | 0.69 (63) | 0.896 |
|  |  | **Others** | 0.32 (53) | 0.31 (29) | (0.990) |
| ***CYP!1B2*** | **rs1799998/ rs10087214** | **Haplotype** | **(N = 320)** | **(N = 188)** |  |
|  |  | **G-A** | 0.05 (15) | 0.08 (15) | 0.085 |
|  |  | **Others** | 0.95 (305) | 0.92 (173) | (0.998) |
|  |  | **Diplotype** | **(N = 160)** | **(N = 194)** |  |
|  |  | **GG-AA** | 0.04 (7) | 0.06 (6) | 0.483 |
|  |  | **Others** | 0.96 (153) | 0.94 (88) | (0.999) |
| ***ADRB2*** | **rs1042713/ rs1042714** | **Haplotype** | **(N = 330)** | **(N = 190)** |  |
|  |  | **G-C** | 0.49 (161) | 0.47 (90) | 0.785 |
|  |  | **Others** | 0.51 (169) | 0.53 (100) | (0.999) |
|  |  | **Diplotype** | **(N = 165)** | **(N = 95)** |  |
|  |  | **GG-CC** | 0.20 (33) | 0.23 (22) | 0.548 |
|  |  | **Others** | 0.80 (132) | 0.77 (73) | (0.99) |

HT, hypertensive; NT, normotensive.
