# Supplementary material for: Genetic Association of the Renin-Angiotensin-Aldosterone System with hypertension among the Malays and their adaptation to climate change
Source: PLoS One. 2026 Apr 15;21(4):e0346614. doi: 10.1371/journal.pone.0346614 (PMC13082722; doi:10.1371/journal.pone.0346614)
Supplement: S12 Table — (DOCX) [file pone.0346614.s012.docx]

**S12 Table. Genotypes for the *AGT*, *CYP11B2* and *ADRB2* variants and the changes of mean systolic blood pressure (SBP), diastolic blood pressure (DBP) and mean arterial pressure (MAP) in HT females age 50 years old and above.**

| **Gene** | **rsID#** | **Genotype** | **N** | **SBP**  **(Mean/ SD)** | **DBP (Mean/SD)** | **MAP (Mean/SD)** |
| --- | --- | --- | --- | --- | --- | --- |
| ***AGT*** | **rs699** | **AA** | 2 | 154.5 ± 12.0 | 76.7 ± 10.2 | 102.6 ± 10.8 |
|  |  | **AG** | 17 | 157.9 ± 11.6 | 83.4 ± 10.9 | 108.3 ± 9.3 |
|  |  | **GG** | 59 | 153.4 ± 12.3 | 85.7 ± 10.6 | 108.3 ± 9.1 |
|  | **rs5051** | **TT** | 58 | 152.7 ± 12.0 | 85.8 ± 10.7 | 108.1 ± 9.1 |
|  |  | **TC** | 15 | 160.4 ± 10.9 | 81.9 ± 12.0 | 108.1 ± 10.5 |
|  |  | **CC** | 1 | 163 | 84 | 110.3 |
| ***CYP11B2*** | **rs1799998** | **GG** | 2 | 152.5 ± 6.3 | 70.2 ± 0.3 | 97.6 ± 2.3 |
|  |  | **GA** | 32 | 158.6 ± 14.0 | 85.7 ± 12.5 | 110.0 ± 10.8 |
|  |  | **AA** | 37 | 151.5 ± 9.8 | 85.0 ± 9.6 | 107.2 ± 7.8 |
|  | **rs10087214** | **GG** | 42 | 151.8 ± 9.6 | 85.1 ± 9.7 | 107.3 ± 7.8 |
|  |  | **GA** | 35 | 157.7 ± 14.3 | 85.4± 11.7 | 109.5 ± 10.4 |
|  |  | **AA** | 1 | 148 | 70 | 96 |
| ***ADRB2*** | **rs1042713** | **GG** | 27 | 155.9 ± 12.9 | 85.3 ± 14.1 | 108.8 ± 11.7 |
|  |  | **GA** | 32 | 152.8 ± 11.2 | 85.3 ± 8.0 | 107.8 ± 7.2 |
|  |  | **AA** | 19 | 154.9 ± 11.4 | 84.1 ± 9.2 | 107.7 ± 8.2 |
|  | **rs1042714** | **CC** | 62 | 153.7 ± 12.3 | 83.6 ± 10.6 | 107.0 ± 9.0 |
|  |  | **CG** | 14 | 158.4 ± 11.6 | 91.5 ± 9.4 | 113.8 ± 8.3 |
|  |  | **GG** | - | - | - | - |

SBP, systolic blood pressure; DBP, diastolic blood pressure; MAP, mean arterial pressure.
