# Supplementary material for: Genetic Association of the Renin-Angiotensin-Aldosterone System with hypertension among the Malays and their adaptation to climate change
Source: PLoS One. 2026 Apr 15;21(4):e0346614. doi: 10.1371/journal.pone.0346614 (PMC13082722; doi:10.1371/journal.pone.0346614)
Supplement: S13 Table — Females age 50 years and above that carried AGT rs5051-TT or CYP11B2 rs1799998-AA had average lower SBP as opposed to those who carried other genotypes; whereas carriers of ADRB2 rs1047714-CC had average lower MAP as opposed to those who carried outer genotypes. (DOCX) [file pone.0346614.s013.docx]

**S13 Table. Association of *AGT*, *CYP11B2* and *ADRB2* variants of the HT females age 50 years and above, and the changes of mean systolic blood pressure (SBP), diastolic blood pressure (DBP) and mean arterial pressure (MAP).** Females age 50 years and above that carried *AGT* rs5051-TT or *CYP11B2* rs1799998-AA had average lower SBP as opposed to those who carried other genotypes; whereas carriers of *ADRB2* rs1047714-CC had average lower MAP as opposed to those who carried outer genotypes.

| **Gene** | **rsID#** | **Genotype** | **Female ≥ 50 y/o** | | | | | | | **Female ≤ 49 y/o** | | | | | | |  |
| --- | --- | --- | --- | --- | --- | --- | --- | --- | --- | --- | --- | --- | --- | --- | --- | --- | --- |
|  |  |  | **N** | **SBP** | **p-value (SBP)** | **DBP (Mean/SD)** | **p-value (DBP)** | **MAP (Mean/SD)** | **p-value (MAP)** | **N** | **SBP** | **p-value (SBP)** | **DBP (Mean/SD)** | **p-value (DBP)** | **MAP (Mean/SD)** | **p-value (MAP)** | |
|  |  |  |  | **(Mean/ SD)** |  |  |  |  |  |  | **(Mean/ SD)** |  |  |  |  |  |  |
| ***AGT*** | **rs699** | GG | 59 | 153.4 ± 12.3 | 0.202 | 82.7 ± 10.8 | 0.291 | 107.7 ± 9.3 | 0.802 | 39 | 149.7 ± 182 | 0.461 | 92.8 ± 9.6 | 0.441 | 111.8 ± 11.6 | 0.963 | |
|  |  | AA + AG | 19 | 157.5 ± 11.4 |  | 85.7 ± 10.6 |  | 108.3 ± 9.1 |  | 16 | 145.7 ± 16.9 |  | 95.0 ± 9.2 |  | 111.9 ± 11.0 |  |  |
|  | **rs5051** | TT | 58 | 152.7 ± 12.0 | ***0.022**** | 85.8 ± 10.7 | 0.236 | 108.1 ± 9.1 | 0.958 | 39 | 149.4 ± 18.2 | 0.579 | 92.7 ± 9.6 | 0.354 | 111.6 ± 11.6 | 0.82 | |
|  |  | TC + CC | 16 | 160.5 ± 10.5 |  | 82.1 ± 11.6 |  | 108.2 ± 10.1 |  | 16 | 146.4 ± 17.1 |  | 95.3 ± 9.1 |  | 112.4 ± 10.9 |  |  |
| ***CYP11B2*** | **rs1799998** | AA | 37 | 151.5 ± 9.8 | ***0.020**** | 85.0 ± 9.6 | 0.937 | 107.2 ± 7.8 | 0.357 | 27 | 154.8 ± 20.9 | ***0.012**** | 94.6 ± 11.8 | 0.382 | 114.7 ± 14.1 | 0.075 | |
|  |  | GG + GA | 34 | 158.2 ± 13.7 |  | 84.8 ± 12.7 |  | 109.3 ± 10.9 |  | 27 | 142.7 ± 11.8 |  | 92.3 ± 6.6 |  | 109.1 ± 7.0 |  |  |
|  | **rs10087214** | GG | 42 | 151.8 ± 9.6 | 0.051 | 85.1 ± 9.7 | 0.964 | 107.3 ± 7.8 | 0.394 | 5 | 142.9 ± 3.8 | 0.46 | 89.4 ± 5.7 | 0.317 | 107.2 ±4.2 | 0.342 | |
|  |  | GA+ AA | 36 | 157.4 ± 14.1 |  | 84.9 ± 11.8 |  | 109.1 ± 10.5 |  | 50 | 149.1 ± 18.6 |  | 93.9 ± 9.7 |  | 112.3 ± 11.7 |  |  |
| ***ADRB2*** | **rs1042713** | GG | 27 | 155.9 ± 13.9 | 0.432 | 85.3 ± 14.1 | 0.895 | 108.8 ± 11.7 | 0.684 | 15 | 155.2 ± 25.0 | 0.092 | 97.9 ± 11.7 | 0.082 | 117.0 ± 15.6 | 0.115 | |
|  |  | GA + AA | 51 | 153.6 ± 11.2 |  | 84.9 ± 8.4 |  | 107.8 ± 7.5 |  | 40 | 146.1 ± 13.8 |  | 91.8 ± 8.0 |  | 109.3 ± 8.7 |  |  |
|  | **rs1042714** | CC | 62 | 153.7 ± 12.3 | 0.196 | 83.6 ± 10.6 | ***0.012**** | 107.0 ± 9.0 | ***0.011**** | 47 | 147.1 ± 17.3 | 0.075 | 92.5 ± 9.0 | 0.05 | 110.7 ± 10.7 | ***0.042**** | |
|  |  | CG + GG | 14 | 158.4 ± 11.6 |  | 91.5 ± 9.4 |  | 113.8 ± 8.3 |  | 7 | 160.0 ± 19.3 |  | 100.1 ± 11.6 |  | 120.1 ± 13.4 |  |  |
