# Supplementary material for: Genetic Association of the Renin-Angiotensin-Aldosterone System with hypertension among the Malays and their adaptation to climate change
Source: PLoS One. 2026 Apr 15;21(4):e0346614. doi: 10.1371/journal.pone.0346614 (PMC13082722; doi:10.1371/journal.pone.0346614)
Supplement: S14 Table — CYP11B2 rs1799998/ rs10087214 AA-GG diplotype had significantly lower SBP as opposed to other diplotypes. AGT rs699/rs5051 G-T haplotype had significantly lower SBP as opposed to other haplotypes, however, the effect of this haplotype is small, by lowering BP measurement only 1 mmHg. (DOCX) [file pone.0346614.s014.docx]

**S14 Table. Association of *CYP11B2* haplotypes and diplotypes of the HT females age 50 years and above, and the changes of mean systolic blood pressure (SBP), diastolic blood pressure (DBP) and mean arterial pressure (MAP).** *CYP11B2* rs1799998/ rs10087214 AA-GG diplotype had significantly lower SBP as opposed to other diplotypes. *AGT* rs699/rs5051 G-T haplotype had significantly lower SBP as opposed to other haplotypes, however, the effect of this haplotype is small, by lowering BP measurement only 1 mmHg.

| **Gene** | **rsID#** |  | **Female ≥ 50 y/o** | | | | | | | | **Female ≤ 49 y/o** | | | | | | | |  |
| --- | --- | --- | --- | --- | --- | --- | --- | --- | --- | --- | --- | --- | --- | --- | --- | --- | --- | --- | --- |
|  |  |  | **N** | **SBP (Mean/SD)** | **p-value (SBP)** | **DBP (Mean/SD)** | **p-value (DBP)** | **MAP (Mean/SD)** | **p-value (MAP)** | **N** | | **SBP (Mean/SD)** | **p-value (SBP)** | **DBP (Mean/SD)** | **p-value (DBP)** | **MAP (Mean/SD)** | **p-value (MAP)** |  |  |
|  |  |  |  |  |  |  |  |  |  |  |  |  |  |  |  |  |  |  | |
| ***AGT*** | **rs699 / rs5051** | **Haplotype** |  |  |  |  |  |  |  |  | |  |  |  |  |  |  |  | |
|  |  | G-T | 114 | 152.9 ± 12.1 | ***0.007**** | 85.7 ± 10.7 | 0.117 | 108.1 ± 9.1 | 0.948 | 77 | | 149.6 ± 18.1 | 0.338 | 92.8 ± 9.6 | 0.27 | 111.7 ± 11.6 | 0.906 |  | |
|  |  | Others | 34 | 153.9 ± 10.9 |  | 82.4 ± 11.2 |  | 108.0 ± 9.7 |  | 33 | | 146.0 ± 16.5 |  | 95.0 ± 8.9 |  | 112.0 ± 10.6 |  |  | |
|  |  | **Diplotype** |  |  |  |  |  |  |  |  | |  |  |  |  |  |  |  | |
|  |  | GG-TT | 55 | 153.0 ± 12.2 | 0.081 | 85.7 ± 10.9 | 0.296 | 108.2 ± 9.3 | 0.948 | 38 | | 149.5 ± 18.4 | 0.546 | 92.8 ± 9.7 | 0.44 | 111.7 ± 11.7 | 0.906 |  | |
|  |  | Others | 19 | 158.6 ± 11.2 |  | 82.7 ± 10.8 |  | 108.0 ± 9.3 |  | 17 | | 146.3 ± 16.6 |  | 95.0 ± 8.9 |  | 112.1 ± 10.6 |  |  | |
| ***CYP11B2*** | **rs1799998/ rs10087214** | **Haplotype** |  |  |  |  |  |  |  |  | |  |  |  |  |  |  |  | |
|  |  | A-G | 76 | 151.8 ± 9.7 | ***0.003**** | 85.1 ± 9.8 | 0.85 | 107.3 ± 7.9 | 0.258 | 27 | | 154.8 ± 20.9 | ***0.012**** | 94.6 ± 11.8 | 0.382 | 114.7 ± 14.1 | 0.075 |  | |
|  |  | Others | 66 | 158.1 ± 13.7 |  | 84.7 ± 12.5 |  | 109.2 ± 10.8 |  | 27 | | 142.7 ± 11.8 |  | 92.3 ± 6.6 |  | 109.1 ± 7.0 |  |  | |
|  |  | **Diplotype** |  |  |  |  |  |  |  |  | |  |  |  |  |  |  |  | |
|  |  | AA-GG | 37 | 151.5 ± 9.8 | ***0.020**** | 85.0 ± 9.6 | 0.937 | 107.2 ± 7.8 | 0.357 | 5 | | 142.9 ± 3.8 | 0.444 | 89.4 ± 5.7 | 0.318 | 107.2 ± 4.2 | 0.335 |  | |
|  |  | Others | 34 | 158.2 ± 13.7 |  | 84.8 ± 12.7 |  | 109.3 ± 10.9 |  | 49 | | 149.4 ± 18.7 |  | 93.9 ± 9.8 |  | 112.4 ± 11.8 |  |  | |
| ***ADRB2*** | **rs1042713/ rs1042714** | **Haplotype** |  |  |  |  |  |  |  |  | |  |  |  |  |  |  |  | |
|  |  | G-C | 77 | 154.1 ± 13.0 | 0.631 | 84.3 ± 12.0 | 0.397 | 107.6 ± 10.0 | 0.383 | 77 | | 149.6 ± 18.2 | 0.338 | 92.8 ± 9.6 | 0.27 | 111.7 ± 11.6 | 0.906 |  | |
|  |  | Others | 75 | 155.1 ± 11.4 |  | 86.8 ± 9.3 |  | 108.9 ± 8.2 |  | 33 | | 146.0 ± 16.5 |  | 95.0 ± 8.9 |  | 112.0 ± 10.6 |  |  | |
|  |  | **Diplotype** |  |  |  |  |  |  |  |  | |  |  |  |  |  |  |  | |
|  |  | GG-CC | 18 | 153.7 ± 14.7 | 0.722 | 81.2 ± 14.4 | 0.175 | 105.3 ± 11.8 | 0.13 | 9 | | 152.6 ± 27.6 | 0.492 | 95.1 ± 11.7 | 0.58 | 114.3 ± 16.5 | 0.502 |  | |
|  |  | Others | 58 | 154.9 ± 11.5 |  | 86.3 ± 9.2 |  | 109.1 ± 8.2 |  | 45 | | 148.0 ± 15.6 |  | 93.2 ± 9.2 |  | 111.4 ± 10.3 |  |  | |
