# Supplementary material for: Genetic Association of the Renin-Angiotensin-Aldosterone System with hypertension among the Malays and their adaptation to climate change
Source: PLoS One. 2026 Apr 15;21(4):e0346614. doi: 10.1371/journal.pone.0346614 (PMC13082722; doi:10.1371/journal.pone.0346614)
Supplement: S15 Table — Data was predominantly obtained from PGG.SNV database (https://www.pggsnv.org/index.html), 1000 Genomes database, Singapore Genome Variation Project (SGVP) [1], Liu et al (2015) [2] for NGO data, Yew et al (2018) [3] for NB data, and 230 hypertension subjects genotyped with Illumina 660W and OminExpress [4]. ‘-‘, data not available; SBP, systolic blood pressure; DBP, diastolic blood pressure; MAP, mean arterial pressure; BMI, body mass index. (DOCX) [file pone.0346614.s015.docx]

**S15 Table. Allele and genotype frequencies of the global populations.** Data was predominantly obtained from PGG.SNV database (<https://www.pggsnv.org/index.html>), 1000 Genomes database, Singapore Genome Variation Project (SGVP) [1] , Liu et al (2015) [2] for NGO data, Yew et al (2018) [3] for NB data, and 230 hypertension subjects genotyped with Illumina 660W and OminExpress [4]. ‘-‘, data not available; SBP, systolic blood pressure; DBP, diastolic blood pressure; MAP, mean arterial pressure; BMI, body mass index.

| **Population** | **Region** | **\|latitude\|** | | **BMI (male)** | | **Mean SBP (male)** | | **Mean DBP (male)** | | **MAP (male)** | | **rs1799998** | | | | **rs10087214** | | | | **rs699** | | | | **rs5051** | | | |  |  |  |  |
| --- | --- | --- | --- | --- | --- | --- | --- | --- | --- | --- | --- | --- | --- | --- | --- | --- | --- | --- | --- | --- | --- | --- | --- | --- | --- | --- | --- | --- | --- | --- | --- |
|  |  |  |  |  |  |  |  |  |  |  |  | **Alternative allele freq (A/T)** | **Ancetral allele freq (C/G)** | **Alternative geno freq (AA)** | **Ancestral geno freq (GG)** | **Alternative allele freq (A/T)** | **Ancetral allele freq (C/G)** | **Alternative geno freq (AA)** | **Ancestral geno freq (GG)** | **Alternative allele freq (A/T)** | **Ancestral allele freq (G/C)** | **Alternative geno freq (AA)** | **Ancestral geno freq (GG)** | **Ancestral allele freq (T/A)** | **Alternative allele freq (C/G)** | **Ancestral geno freq (TT)** | **Alternative geno freq (GG)** |  |  |  |  |
| Khwe | Africa | 28 | | - | | 129.42 | | 79.61 | | 96.21 | | 0.625 | 0.375 | 0.375 | 0.125 | 0.281 | 0.719 | 0.063 | 0.500 | 0.031 | 0.969 | 0.000 | 0.938 | 1.000 | 0.000 | 1.000 | 0.000 |  |  |  |  |
| Luhya_Kenya | Africa | 0.37 | | 22.22533 | | 129.78 | | 76.96 | | 94.57 | | 0.798 | 0.202 | 0.626 | 0.030 | 0.202 | 0.798 | 0.030 | 0.626 | 0.111 | 0.889 | 0.020 | 0.798 | 0.897 | 0.103 | 0.809 | 0.014 |  |  |  |  |
| Baka_Cameroon | Africa | 6 | | 23.66 | | 130.13 | | 78.50 | | 95.71 | | 0.802 | 0.198 | 0.621 | 0.017 | - | - | - | - | 0.017 | 0.983 | 0.000 | 0.966 | 0.983 | 0.017 | 0.966 | 0.000 |  |  |  |  |
| Mande_SierraLeone | Africa | 8.5 | | 21.91 | | 131.26 | | 78.67 | | 96.20 | | 0.806 | 0.194 | 0.635 | 0.024 | 0.171 | 0.829 | 0.024 | 0.682 | 0.024 | 0.976 | 0.000 | 0.953 | 0.976 | 0.024 | 0.953 | 0.000 |  |  |  |  |
| Esan_Nigeria | Africa | 10 | | 22.65 | | 125.06 | | 77.30 | | 93.22 | | 0.864 | 0.136 | 0.747 | 0.020 | 0.136 | 0.864 | 0.020 | 0.747 | 0.066 | 0.934 | 0.010 | 0.879 | 0.960 | 0.041 | 0.929 | 0.010 |  |  |  |  |
| Gambian | Africa | 13.3 | | 23.56 | | 132.05 | | 80.14 | | 97.44 | | 0.757 | 0.243 | 0.575 | 0.062 | 0.243 | 0.757 | 0.062 | 0.575 | 0.066 | 0.934 | 0.009 | 0.876 | 0.951 | 0.049 | 0.903 | 0.000 |  |  |  |  |
| Mozabite | Africa | 32 | | 22.07 | | 133.58 | | 78.17 | | 96.64 | | - | - | - | - | 0.296 | 0.704 | 0.074 | 0.481 | 0.500 | 0.500 | 0.222 | 0.222 | - | - | - | - |  |  |  |  |
| Peruvian | America | 10 | | 26.37 | | 124.23 | | 73.12 | | 90.16 | | 0.429 | 0.571 | 0.235 | 0.376 | 0.571 | 0.429 | 0.376 | 0.235 | 0.253 | 0.747 | 0.059 | 0.553 | 0.753 | 0.247 | 0.553 | 0.047 |  |  |  |  |
| Colombian | America | 6.17 | | 25.99 | | 126.40 | | 77.86 | | 94.04 | | 0.580 | 0.420 | 0.372 | 0.213 | - | - | - | - | 0.452 | 0.548 | 0.223 | 0.319 | 0.553 | 0.447 | 0.319 | 0.213 |  |  |  |  |
| PuertoRican | America | 18.15 | | 28.47 | | 125.51 | | 78.74 | | 94.33 | | 0.577 | 0.423 | 0.327 | 0.173 | 0.423 | 0.577 | 0.173 | 0.327 | 0.414 | 0.587 | 0.183 | 0.356 | 0.615 | 0.385 | 0.394 | 0.163 |  |  |  |  |
| Mexican | America | 32.74 | | 27.64 | | 127.55 | | 78.43 | | 94.80 | | 0.443 | 0.557 | 0.320 | 0.207 | - | - | - | - | 0.305 | 0.695 | 0.094 | 0.484 | 0.687 | 0.313 | 0.460 | 0.087 |  |  |  |  |
| Tajiks | CentralAsiaSiberia | 37.4 | | 26.01 | | 130.60 | | 80.95 | | 97.50 | | - | - | - | - | 0.525 | 0.475 | 0.250 | 0.200 | - | - | - | - | - | - | - | - |  |  |  |  |
| Uzbeks | CentralAsiaSiberia | 41.3 | | 26.49 | | 131.20 | | 82.62 | | 98.81 | | - | - | - | - | 0.375 | 0.625 | 0.208 | 0.458 | - | - | - | - | - | - | - | - |  |  |  |  |
| Russian | CentralAsiaSiberia | 61 | | 25.99 | | 130.33 | | 82.15 | | 98.21 | | - | - | - | - | 0.540 | 0.460 | 0.280 | 0.200 | 0.620 | 0.380 | 0.360 | 0.120 | - | - | - | - |  |  |  |  |
| Taiwan.Han | EastAsia | 25.03 | | 25.14 | | 121.53 | | 76.08 | | 91.23 | | - | - | - | - | 0.281 | 0.719 | 0.081 | 0.520 | - | - | - | - | - | - | - | - |  |  |  |  |
| Japanese | EastAsia | 35.41 | | 23.68 | | 125.57 | | 78.08 | | 93.91 | | 0.620 | 0.380 | 0.375 | 0.135 | 0.380 | 0.620 | 0.135 | 0.375 | 0.149 | 0.851 | 0.010 | 0.712 | 0.805 | 0.195 | 0.625 | 0.015 |  |  |  |  |
| Han.Beijing | EastAsia | 39 | | 24.28 | | 126.11 | | 78.48 | | 94.36 | | 0.731 | 0.269 | 0.500 | 0.037 | 0.299 | 0.701 | 0.091 | 0.493 | 0.208 | 0.792 | 0.058 | 0.642 | 0.785 | 0.215 | 0.625 | 0.054 |  |  |  |  |
| Mongola | EastAsia | 47 | | 26.05 | | 133.38 | | 81.45 | | 98.76 | | - | - | - | - | - | - | - | - | 0.200 | 0.800 | 0.000 | 0.600 | - | - | - | - |  |  |  |  |
| SriLankan.Tamil | SouthAsia | 8.58 | | 22.64 | | 127.63 | | 75.37 | | 92.79 | | 0.672 | 0.328 | 0.480 | 0.137 | 0.304 | 0.696 | 0.118 | 0.510 | 0.319 | 0.681 | 0.098 | 0.461 | 0.686 | 0.314 | 0.471 | 0.098 |  |  |  |  |
| Indian.Telugu | SouthAsia | 14.96 | | 21.81 | | 127.26 | | 80.53 | | 96.11 | | 0.593 | 0.407 | 0.373 | 0.186 | 0.392 | 0.608 | 0.167 | 0.382 | 0.378 | 0.623 | 0.147 | 0.392 | 0.623 | 0.378 | 0.392 | 0.147 |  |  |  |  |
| Bengali | SouthAsia | 23.7 | | 21.40 | | 119.53 | | 77.12 | | 91.26 | | 0.692 | 0.308 | 0.512 | 0.128 | 0.308 | 0.692 | 0.128 | 0.512 | 0.320 | 0.680 | 0.093 | 0.453 | 0.680 | 0.320 | 0.453 | 0.093 |  |  |  |  |
| Punjabi | SouthAsia | 31.5 | | 22.70 | | 129.04 | | 83.83 | | 98.90 | | 0.578 | 0.422 | 0.354 | 0.198 | 0.406 | 0.594 | 0.188 | 0.375 | 0.391 | 0.609 | 0.177 | 0.396 | 0.620 | 0.380 | 0.406 | 0.167 |  |  |  |  |
| Kinh_Vietnam | SoutheastAsia | 21.2 | | 21.97 | | 127.25 | | 77.06 | | 93.79 | | 0.737 | 0.263 | 0.556 | 0.081 | 0.263 | 0.737 | 0.081 | 0.556 | 0.121 | 0.879 | 0.010 | 0.768 | 0.879 | 0.121 | 0.768 | 0.010 |  |  |  |  |
| Spore_Malay | SoutheastAsia | 1.29 | | 24.40 | | 122.11 | | 74.82 | | 90.58 | | 0.781 | 0.219 | 0.594 | 0.031 | 0.193 | 0.807 | 0.021 | 0.635 | 0.182 | 0.818 | 0.031 | 0.667 | 0.813 | 0.188 | 0.656 | 0.031 |  |  |  |  |
| P.Msia_Malay | SoutheastAsia | 3.1412 | | 25.40 | | 115.80 | | 86.80 | | 96.47 | | 0.760 | 0.240 | 0.600 | 0.080 | 0.240 | 0.760 | 0.080 | 0.600 | 0.180 | 0.820 | 0.040 | 0.690 | 0.830 | 0.170 | 0.700 | 0.040 |  |  |  |  |
| Bateq | SoutheastAsia | 4 | | 19.20 | | 134.00 | | 85.00 | | 101.33 | | 0.982 | 0.018 | 0.964 | 0.000 | - | - | - | - | 0.200 | 0.800 | 0.000 | 0.714 | 0.857 | 0.143 | 0.714 | 0.000 |  |  |  |  |
| Cambodian | SoutheastAsia | 12 | | 22.15 | | 124.12 | | 75.01 | | 91.38 | | - | - | - | - | 0.350 | 0.650 | 0.200 | 0.500 | 0.150 | 0.850 | 0.000 | 0.700 | - | - | - | - |  |  |  |  |
| Burmese | SoutheastAsia | 21.98 | | 22.32 | | 126.25 | | 75.53 | | 92.43 | | - | - | - | - | 0.200 | 0.800 | 0.133 | 0.733 | - | - | - | - | - | - | - | - |  |  |  |  |
| Saudis | WestEurasia | 24.91 | | 28.14 | | 126.18 | | 80.02 | | 95.41 | | - | - | - | - | 0.350 | 0.650 | 0.050 | 0.350 | - | - | - | - | - | - | - | - |  |  |  |  |
| Palestinian | WestEurasia | 32 | | 28.27 | | 126.27 | | 79.61 | | 95.17 | | - | - | - | - | 0.478 | 0.522 | 0.239 | 0.283 | 0.478 | 0.522 | 0.217 | 0.261 | - | - | - | - |  |  |  |  |
| Iberian | WestEurasia | 40 | | - | | 127.20 | | 78.89 | | 94.99 | | 0.547 | 0.453 | 0.299 | 0.206 | 0.453 | 0.547 | 0.206 | 0.299 | 0.575 | 0.425 | 0.355 | 0.206 | 0.439 | 0.561 | 0.215 | 0.336 |  |  |  |  |
| French | WestEurasia | 46 | | 26.07 | | 127.04 | | 77.04 | | 93.71 | | - | - | - | - | 0.482 | 0.518 | 0.179 | 0.214 | 0.679 | 0.321 | 0.500 | 0.143 | - | - | - | - |  |  |  |  |
| Italian | WestEurasia | 46 | | 26.65 | | 127.68 | | 78.89 | | 95.15 | | - | - | - | - | 0.542 | 0.458 | 0.333 | 0.250 | 0.375 | 0.625 | 0.167 | 0.417 | - | - | - | - |  |  |  |  |
| Hungarians | WestEurasia | 47.5 | | 28.40 | | 134.88 | | 82.85 | | 100.19 | | - | - | - | - | 0.316 | 0.684 | 0.158 | 0.526 | - | - | - | - | - | - | - | - |  |  |  |  |
| Germans | WestEurasia | 52.52 | | 27.48 | | 126.32 | | 76.47 | | 93.09 | | - | - | - | - | 0.385 | 0.615 | 0.154 | 0.385 | - | - | - | - | - | - | - | - |  |  |  |  |
| Estonians | WestEurasia | 58.5 | | 27.11 | | 135.47 | | 80.77 | | 99.00 | | 0.534 | 0.466 | 0.276 | 0.208 | - | - | - | - | - | - | - | - | - | - | - | - |  |  |  |  |
| Finnish | WestEurasia | 64 | | 26.55 | | 126.90 | | 76.65 | | 93.40 | | 0.493 | 0.507 | 0.253 | 0.267 | - | - | - | - | 0.571 | 0.429 | 0.303 | 0.162 | 0.430 | 0.570 | 0.176 | 0.316 |  |  |  |  |
| -, data not available | | |  | |  | |  | |  | |  | |  |  |  |  |  |  |  |  |  |  |  |  |  |  |  |  |  |  |  |

| **Population** | **Region** | **\|latitude\|** | **BMI (male)** | **Mean SBP (male)** | **Mean DBP (male)** | **MAP (male)** | **rs1042713** | | | | **rs1042714** | | | |
| --- | --- | --- | --- | --- | --- | --- | --- | --- | --- | --- | --- | --- | --- | --- |
|  |  |  |  |  |  |  | **Ancestral allele freq (G/C)** | **Alternative allele freq (A/T)** | **Ancestral geno freq (GG)** | **Alternative geno freq (AA)** | **Ancestral allele freq (G)** | **Alternative allele freq (C)** | **Ancestral geno freq (GG)** | **Alternative geno freq (CC)** |
| Khwe | Africa | 28 | - | 129.42 | 79.61 | 96.21 | 0.570 | 0.430 | 0.333 | 0.200 | - | - | - | - |
| Luhya_Kenya | Africa | 0.37 | 22.22533 | 129.78 | 76.96 | 94.57 | 0.510 | 0.490 | 0.273 | 0.253 | 0.210 | 0.790 | 0.010 | 0.586 |
| Baka_Cameroon | Africa | 6 | 23.66 | 130.13 | 78.50 | 95.71 | 0.420 | 0.580 | 0.103 | 0.259 | - | - | - | - |
| Mande_SierraLeone | Africa | 8.5 | 21.91 | 131.26 | 78.67 | 96.20 | 0.480 | 0.520 | 0.259 | 0.306 | 0.090 | 0.910 | 0.000 | 0.812 |
| Esan_Nigeria | Africa | 10 | 22.65 | 125.06 | 77.30 | 93.22 | 0.480 | 0.520 | 0.192 | 0.222 | 0.120 | 0.880 | 0.010 | 0.768 |
| Gambian | Africa | 13.3 | 23.56 | 132.05 | 80.14 | 97.44 | 0.470 | 0.530 | 0.248 | 0.310 | 0.120 | 0.880 | 0.009 | 0.876 |
| Mozabite | Africa | 32 | 22.07 | 133.58 | 78.17 | 96.64 | 0.670 | 0.330 | 0.370 | 0.037 | - | - | - | - |
| Peruvian | America | 10 | 26.37 | 124.23 | 73.12 | 90.16 | 0.560 | 0.440 | 0.365 | 0.235 | 0.140 | 0.860 | 0.035 | 0.765 |
| Colombian | America | 6.17 | 25.99 | 126.40 | 77.86 | 94.04 | 0.480 | 0.520 | 0.202 | 0.234 | 0.230 | 0.770 | 0.021 | 0.564 |
| PuertoRican | America | 18.15 | 28.47 | 125.51 | 78.74 | 94.33 | 0.590 | 0.410 | 0.337 | 0.154 | 0.400 | 0.600 | 0.135 | 0.327 |
| Mexican | America | 32.74 | 27.64 | 127.55 | 78.43 | 94.80 | 0.520 | 0.480 | 0.250 | 0.203 | 0.140 | 0.860 | 0.031 | 0.750 |
| Tajiks | CentralAsiaSiberia | 37.4 | 26.01 | 130.60 | 80.95 | 97.50 | 0.470 | 0.530 | 0.200 | 0.250 | 0.290 | 0.710 | 0.192 | 0.615 |
| Uzbeks | CentralAsiaSiberia | 41.3 | 26.49 | 131.20 | 82.62 | 98.81 | 0.690 | 0.310 | 0.458 | 0.083 | - | - | - | - |
| Russian | CentralAsiaSiberia | 61 | 25.99 | 130.33 | 82.15 | 98.21 | 0.740 | 0.260 | 0.560 | 0.080 | - | - | - | - |
| Taiwan.Han | EastAsia | 25.03 | 25.14 | 121.53 | 76.08 | 91.23 | - | - | - | - | - | - | - | - |
| Japanese | EastAsia | 35.41 | 23.68 | 125.57 | 78.08 | 93.91 | 0.560 | 0.440 | 0.317 | 0.202 | 0.060 | 0.940 | 0.010 | 0.894 |
| Han.Beijing | EastAsia | 39 | 24.28 | 126.11 | 78.48 | 94.36 | 0.420 | 0.580 | 0.153 | 0.321 | 0.110 | 0.890 | 0.000 | 0.786 |
| Mongola | EastAsia | 47 | 26.05 | 133.38 | 81.45 | 98.76 | 0.550 | 0.450 | 0.300 | 0.200 | - | - | - | - |
| SriLankan.Tamil | SouthAsia | 8.58 | 22.64 | 127.63 | 75.37 | 92.79 | 0.480 | 0.520 | 0.245 | 0.275 | 0.150 | 0.850 | 0.039 | 0.735 |
| Indian.Telugu | SouthAsia | 14.96 | 21.81 | 127.26 | 80.53 | 96.11 | 0.520 | 0.480 | 0.353 | 0.304 | 0.180 | 0.820 | 0.049 | 0.696 |
| Bengali | SouthAsia | 23.7 | 21.40 | 119.53 | 77.12 | 91.26 | 0.570 | 0.430 | 0.326 | 0.186 | 0.170 | 0.830 | 0.023 | 0.674 |
| Punjabi | SouthAsia | 31.5 | 22.70 | 129.04 | 83.83 | 98.90 | 0.570 | 0.430 | 0.333 | 0.188 | 0.230 | 0.770 | 0.083 | 0.615 |
| Kinh_Vietnam | SoutheastAsia | 21.2 | 21.97 | 127.25 | 77.06 | 93.79 | 0.430 | 0.570 | 0.172 | 0.303 | 0.060 | 0.940 | 0.000 | 0.889 |
| Spore_Malay | SoutheastAsia | 1.29 | 24.40 | 122.11 | 74.82 | 90.58 | 0.520 | 0.480 | 0.219 | 0.188 | 0.090 | 0.910 | 0.000 | 0.813 |
| P.Msia_Malay | SoutheastAsia | 3.1412 | 25.40 | 115.80 | 86.80 | 96.47 | 0.540 | 0.460 | 0.290 | 0.230 | 0.090 | 0.910 | 0.010 | 0.840 |
| Bateq | SoutheastAsia | 4 | 19.20 | 134.00 | 85.00 | 101.33 | 0.580 | 0.420 | 0.250 | 0.083 | 0.040 | 0.960 | 0.000 | 0.917 |
| Cambodian | SoutheastAsia | 12 | 22.15 | 124.12 | 75.01 | 91.38 | 0.800 | 0.200 | 0.600 | 0.000 | - | - | - | - |
| Burmese | SoutheastAsia | 21.98 | 22.32 | 126.25 | 75.53 | 92.43 | 0.330 | 0.670 | 0.000 | 0.500 | - | - | - | - |
| Saudis | WestEurasia | 24.91 | 28.14 | 126.18 | 80.02 | 95.41 | 0.550 | 0.450 | 0.250 | 0.150 | - | - | - | - |
| Palestinian | WestEurasia | 32 | 28.27 | 126.27 | 79.61 | 95.17 | 0.490 | 0.510 | 0.217 | 0.239 | - | - | - | - |
| Iberian | WestEurasia | 40 | - | 127.20 | 78.89 | 94.99 | 0.620 | 0.380 | 0.355 | 0.121 | 0.430 | 0.570 | 0.168 | 0.318 |
| French | WestEurasia | 46 | 26.07 | 127.04 | 77.04 | 93.71 | 0.590 | 0.410 | 0.321 | 0.143 | - | - | - | - |
| Italian | WestEurasia | 46 | 26.65 | 127.68 | 78.89 | 95.15 | 0.540 | 0.460 | 0.250 | 0.167 | - | - | - | - |
| Hungarians | WestEurasia | 47.5 | 28.40 | 134.88 | 82.85 | 100.19 | 0.630 | 0.370 | 0.368 | 0.105 | - | - | - | - |
| Germans | WestEurasia | 52.52 | 27.48 | 126.32 | 76.47 | 93.09 | 0.540 | 0.460 | 0.308 | 0.231 | - | - | - | - |
| Estonians | WestEurasia | 58.5 | 27.11 | 135.47 | 80.77 | 99.00 | 0.530 | 0.470 | 0.333 | 0.267 | - | - | - | - |
| Finnish | WestEurasia | 64 | 26.55 | 126.90 | 76.65 | 93.40 | 0.590 | 0.410 | 0.313 | 0.141 | 0.370 | 0.630 | 0.101 | 0.364 |
| -, data not available | | |  |  |  |  |  |  |  |  |  |  |  |  |
