# Supplementary material for: Genetic Association of the Renin-Angiotensin-Aldosterone System with hypertension among the Malays and their adaptation to climate change
Source: PLoS One. 2026 Apr 15;21(4):e0346614. doi: 10.1371/journal.pone.0346614 (PMC13082722; doi:10.1371/journal.pone.0346614)
Supplement: S16 Table — Significantly different allele and genotype frequencies were observed between African and non-African populations; but not between African and Southeast Asian populations. (DOCX) [file pone.0346614.s016.docx]

**S16 Table.** **Differentiation of allele and genotype frequencies between African populations and the non-African populations.** Significantly different allele and genotype frequencies were observed between African and non-African populations; but not between African and Southeast Asian populations.

| **Region** | **Gene** | **rsID#** | **allele / genotype** | **p-value** |
| --- | --- | --- | --- | --- |
| African vs. non-African | *AGT* | rs699 | G | 2.68 x 10^-3^ |
|  |  |  | GG | 6.87 x 10^-4^ |
|  |  | rs5051 | T | 7.80 x 10^-5^ |
|  |  |  | TT | < 1.00 x 10^-5^ |
|  | *CYP11B2* | rs1799998 | G | 0.015 |
|  |  |  | GG | 6.09 x 10^-3^ |
|  |  | rs10087214 | A | 8.64 x 10^-4^ |
|  |  |  | AA | 7.82 x 10^-4^ |
|  | *ADRB2* | rs1042713 | T | 0.167 |
|  |  |  | TT | 0.3338 |
|  |  | rs1042714 | G | 0.21 |
|  |  |  | GG | 0.1627 |
| African vs Southeast Asia | *AGT* | rs699 | G | 0.628 |
|  |  |  | GG | 0.663 |
|  |  | rs5051 | T | 0.613 |
|  |  |  | TT | 0.844 |
|  | *CYP11B2* | rs1799998 | G | 0.533 |
|  |  |  | GG | 0.476 |
|  |  | rs10087214 | A | 0.243 |
|  |  |  | AA | 0.48 |
|  | *ADRB2* | rs1042713 | T | 0.393 |
|  |  |  | TT | 0.904 |
|  |  | rs1042714 | G | 0.032 |
|  |  |  | GG | - |
