# Supplementary material for: Genetic Association of the Renin-Angiotensin-Aldosterone System with hypertension among the Malays and their adaptation to climate change
Source: PLoS One. 2026 Apr 15;21(4):e0346614. doi: 10.1371/journal.pone.0346614 (PMC13082722; doi:10.1371/journal.pone.0346614)
Supplement: S1 Fig — Weak r2 was observed between the ADRB2-rs1042713 and rs1042714. (DOCX) [file pone.0346614.s017.docx]

**S1 Fig. Linkage disequilibrium between the variants for *AGT* (rs699 and rs5051), *CYP11B2* (rs1799998 and rs10087214) and *ADRB2* (rs1042713 and rs1042714).** Weak r^2^ was observed between the *ADRB2*-rs1042713 and rs1042714.
