# Supplementary material for: Genetic Association of the Renin-Angiotensin-Aldosterone System with hypertension among the Malays and their adaptation to climate change
Source: PLoS One. 2026 Apr 15;21(4):e0346614. doi: 10.1371/journal.pone.0346614 (PMC13082722; doi:10.1371/journal.pone.0346614)
Supplement: S2 Fig — AA genotype exhibited significantly higher SBP, DBP and MAP among the Malay HT males, but no significant difference between the Malay HT females. (DOCX) [file pone.0346614.s018.docx]

**S2 Fig. Effect of *CYP11B2-*rs10082714 variants to the changes of BP in (a) males; (b) females; (c) all.** AA genotype exhibited significantly higher SBP, DBP and MAP among the Malay HT males, but no significant difference between the Malay HT females.
