# Supplementary material for: Genetic Association of the Renin-Angiotensin-Aldosterone System with hypertension among the Malays and their adaptation to climate change
Source: PLoS One. 2026 Apr 15;21(4):e0346614. doi: 10.1371/journal.pone.0346614 (PMC13082722; doi:10.1371/journal.pone.0346614)
Supplement: S3 Fig — The mean BP of the global male populations was retrieved from NCD Risk Factor Collaboration (NCD-RisC; http://ncdrisc.org/index.html). Only the mean BP of males was included for subsequent analysis. (DOCX) [file pone.0346614.s019.docx]

**S3 Fig. Correlation between BP and latitude coordinate.** The mean BP of the global male populations was retrieved from NCD Risk Factor Collaboration (NCD-RisC; <http://ncdrisc.org/index.html>). Only the mean BP of males was included for subsequent analysis.
