# Supplementary material for: Genetic Association of the Renin-Angiotensin-Aldosterone System with hypertension among the Malays and their adaptation to climate change
Source: PLoS One. 2026 Apr 15;21(4):e0346614. doi: 10.1371/journal.pone.0346614 (PMC13082722; doi:10.1371/journal.pone.0346614)

**S5 Fig.** **Genotype frequencies of the risk alleles and genotypes of the SNPs *ADRB2*- rs1042713 and rs1042714 with geographical latitude correlation.** (a) rs1042713; (b) r1042714.

**(a)**


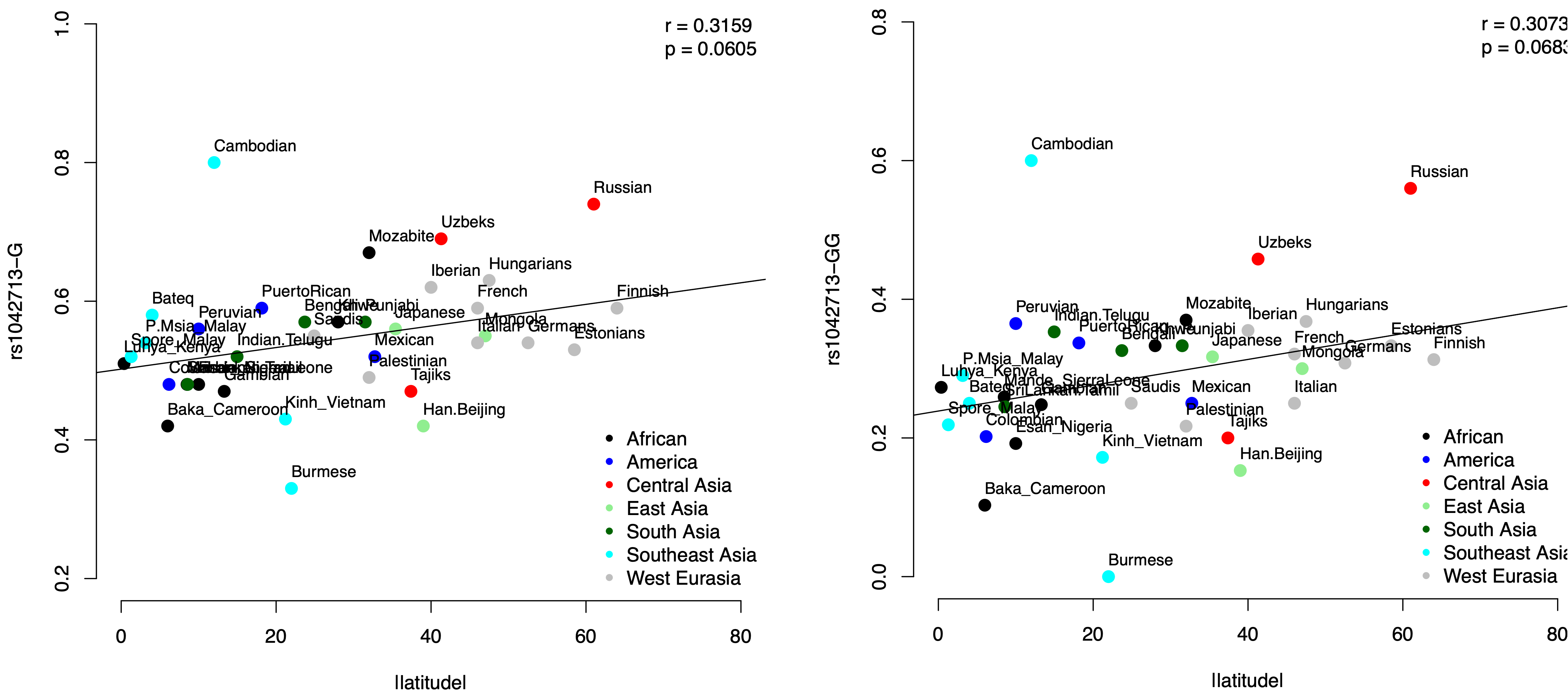


**(b)**


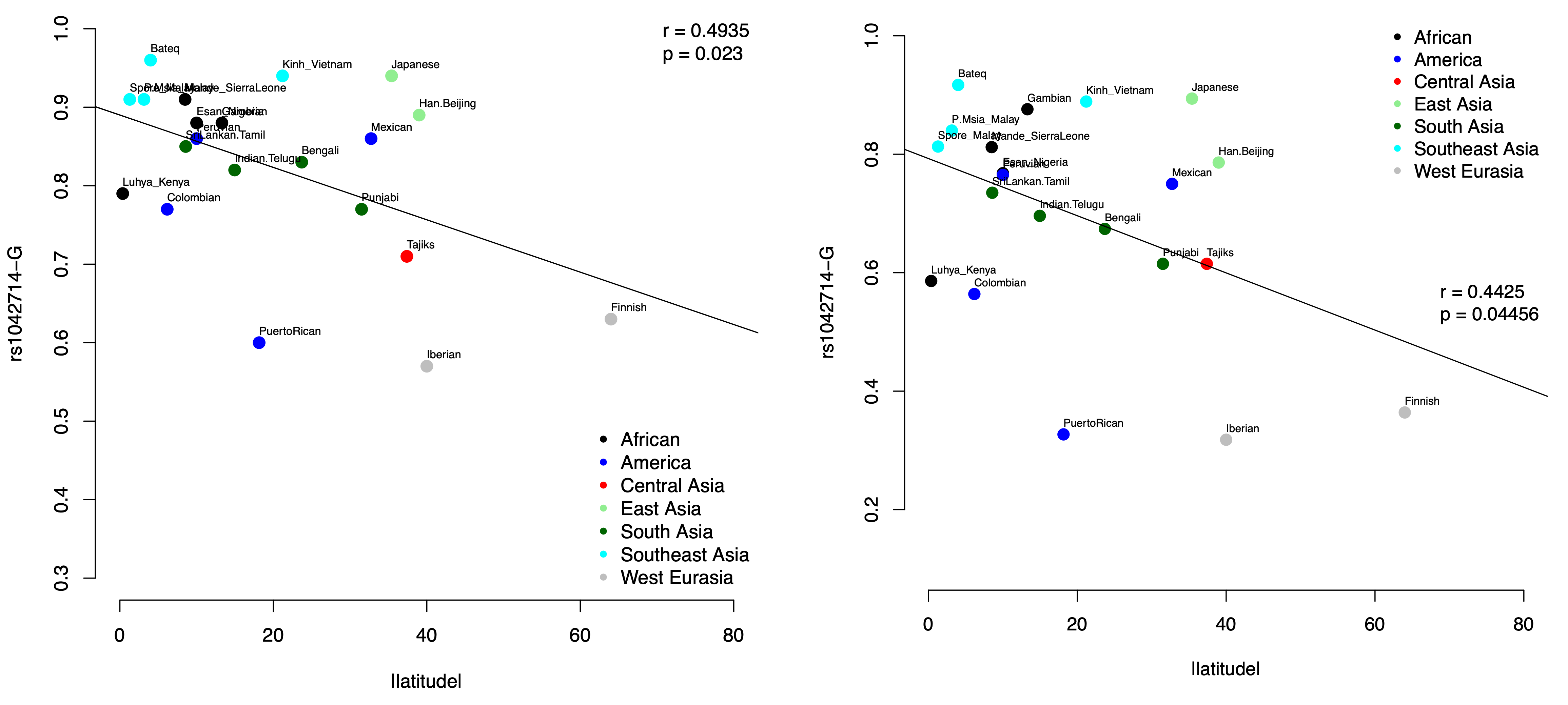

Supplement: S5 Fig — (a) rs1042713; (b) r1042714. (DOCX) [file pone.0346614.s021.docx]
