# Supplementary material for: Genetic Association of the Renin-Angiotensin-Aldosterone System with hypertension among the Malays and their adaptation to climate change
Source: PLoS One. 2026 Apr 15;21(4):e0346614. doi: 10.1371/journal.pone.0346614 (PMC13082722; doi:10.1371/journal.pone.0346614)
Supplement: S6 Fig — (a) AGT-rs699; (b) CYP11B2-rs1799998; (c) CYP11B2-rs10087214; (d) ADRB2-rs1042713; (e) ADRB2-rs1042714. (DOCX) [file pone.0346614.s022.docx]

**S6 Fig. Frequencies of the risk alleles and genotypes of *AGT*, *CYP11B2* and *ADRB2*, and their correlation with body mass index.**(a) *AGT*-rs699; (b) *CYP11B2*-rs1799998; (c) *CYP11B2*-rs10087214; (d) *ADRB2*-rs1042713; (e) *ADRB2*-rs1042714

**a.**


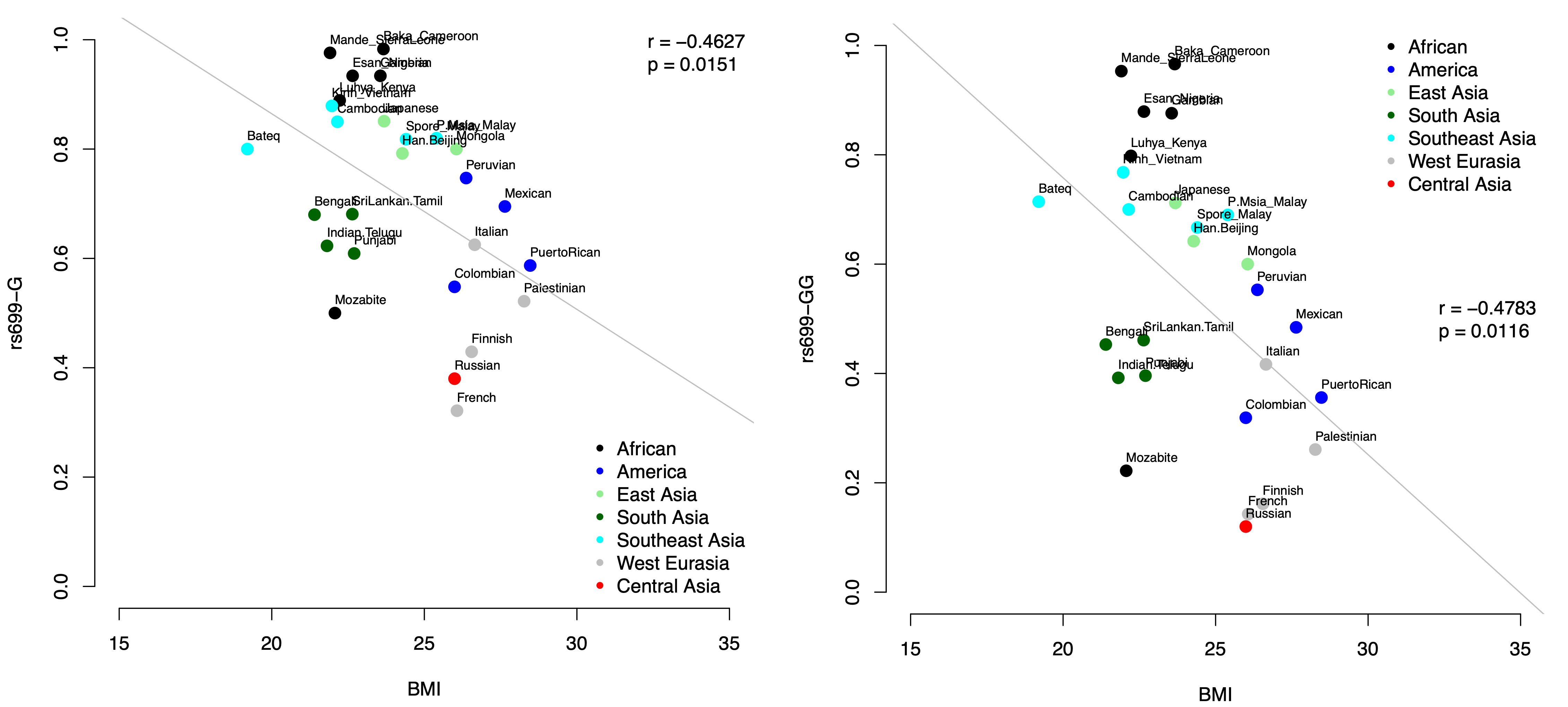


**b.**


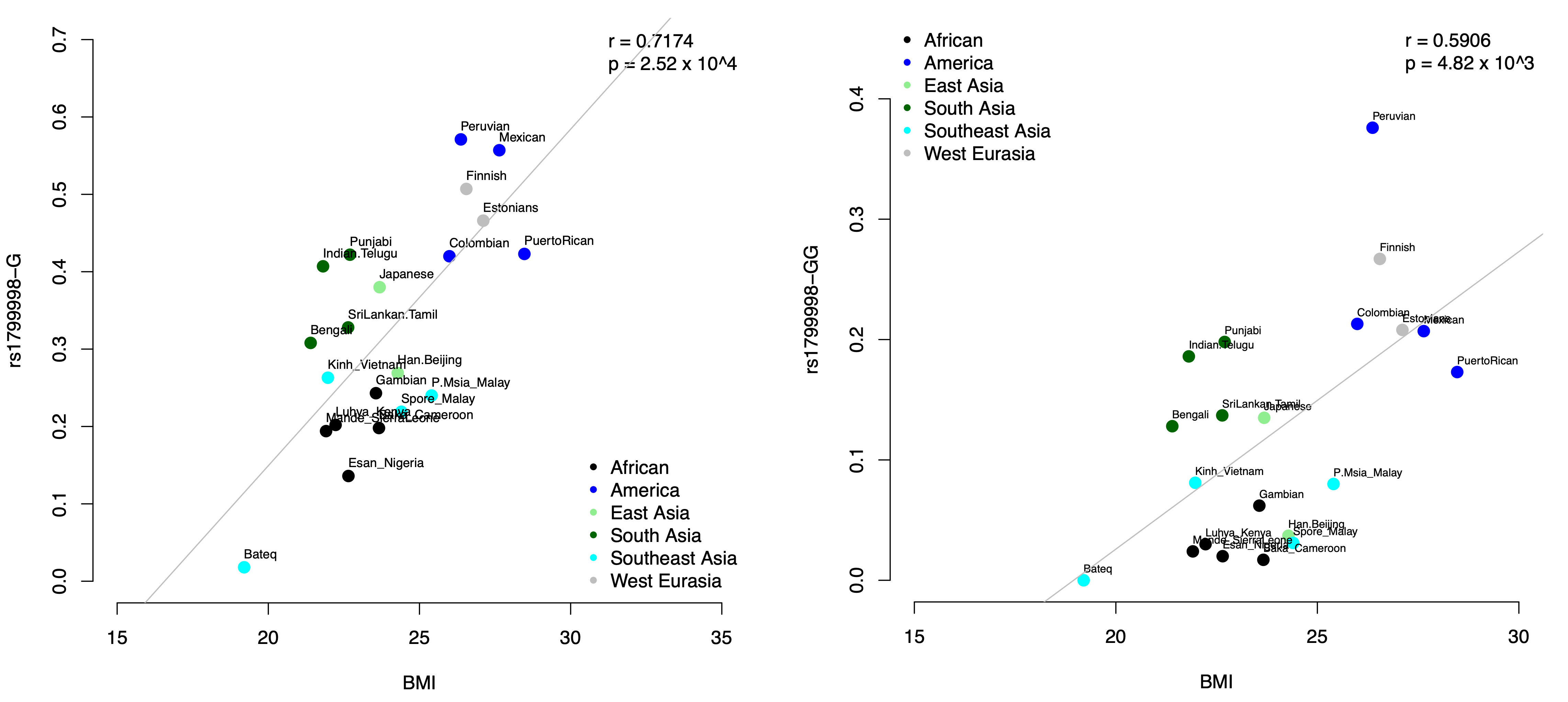


**c.**


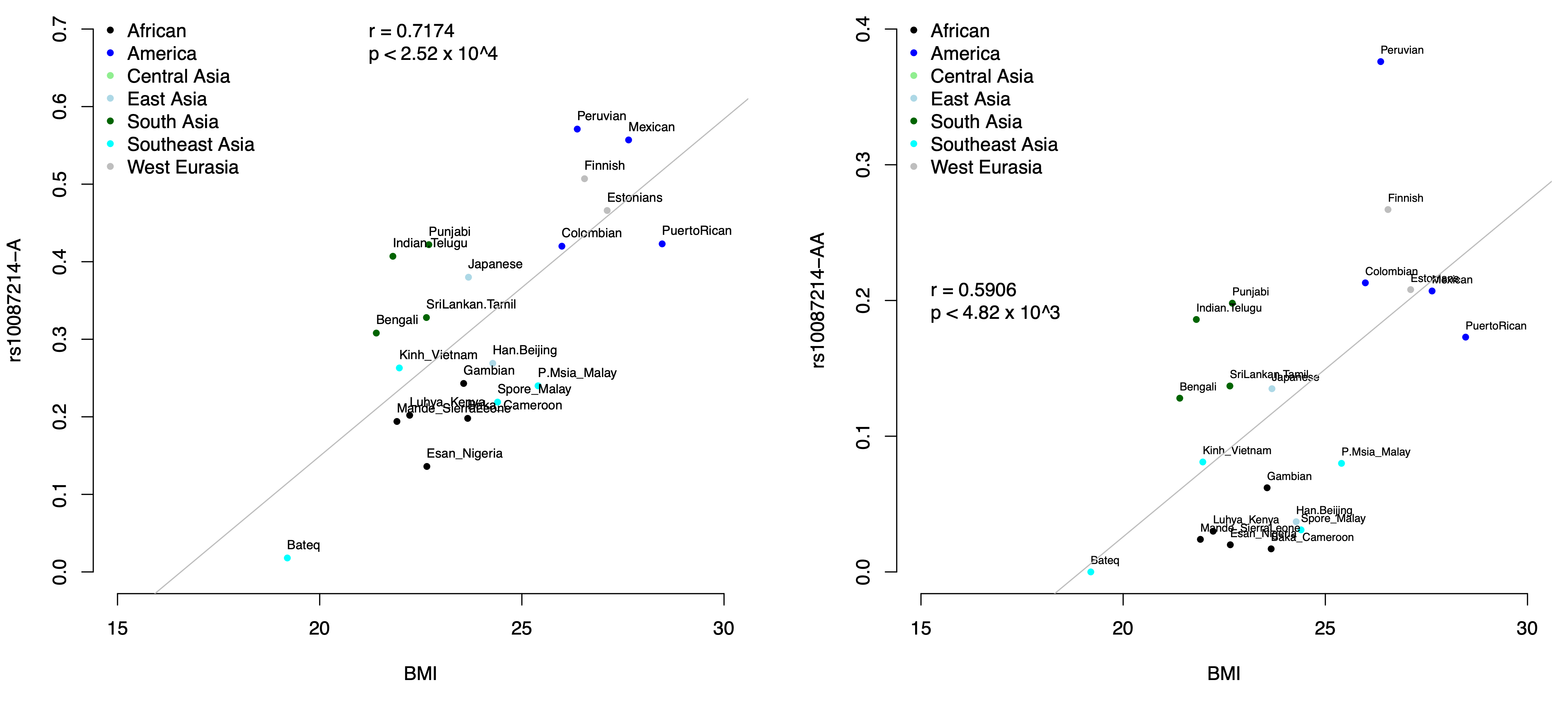


**d.**

**
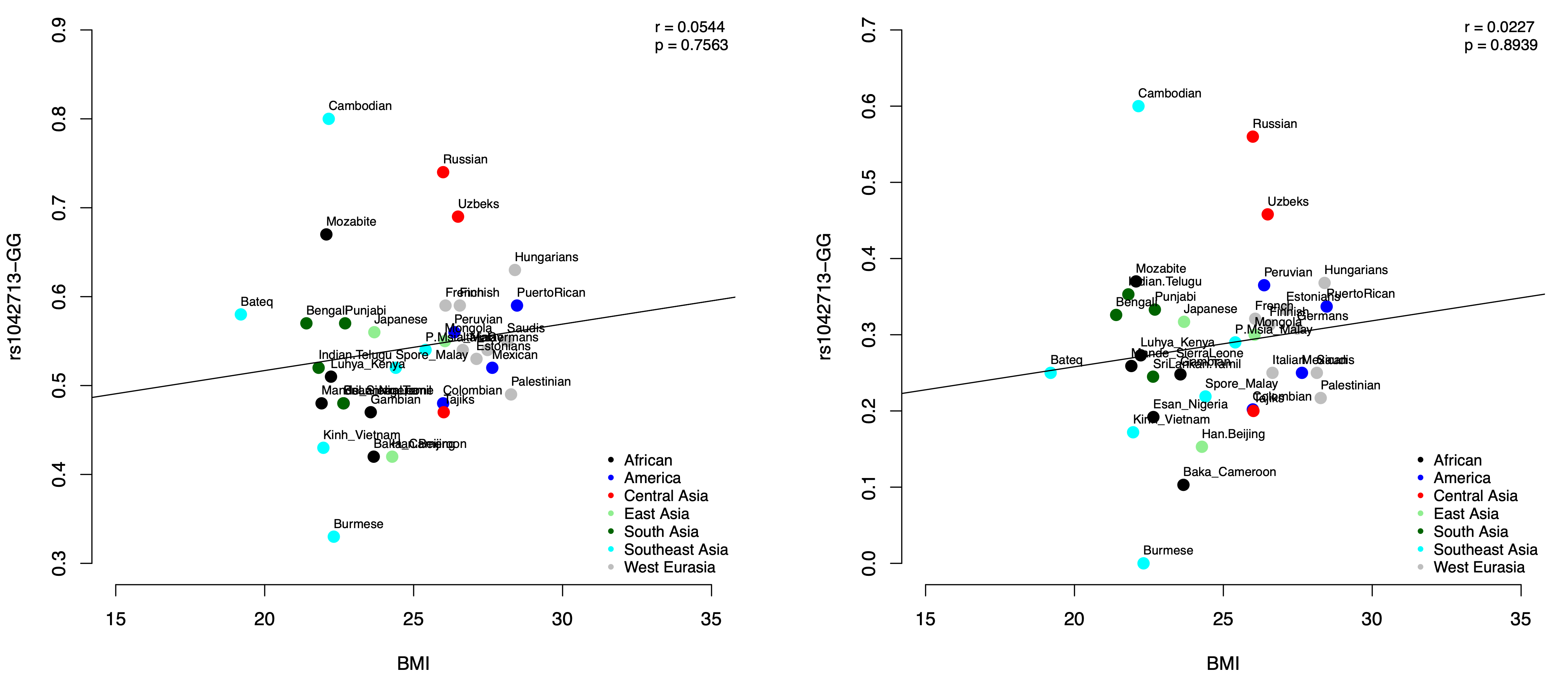
**

**e.**

**
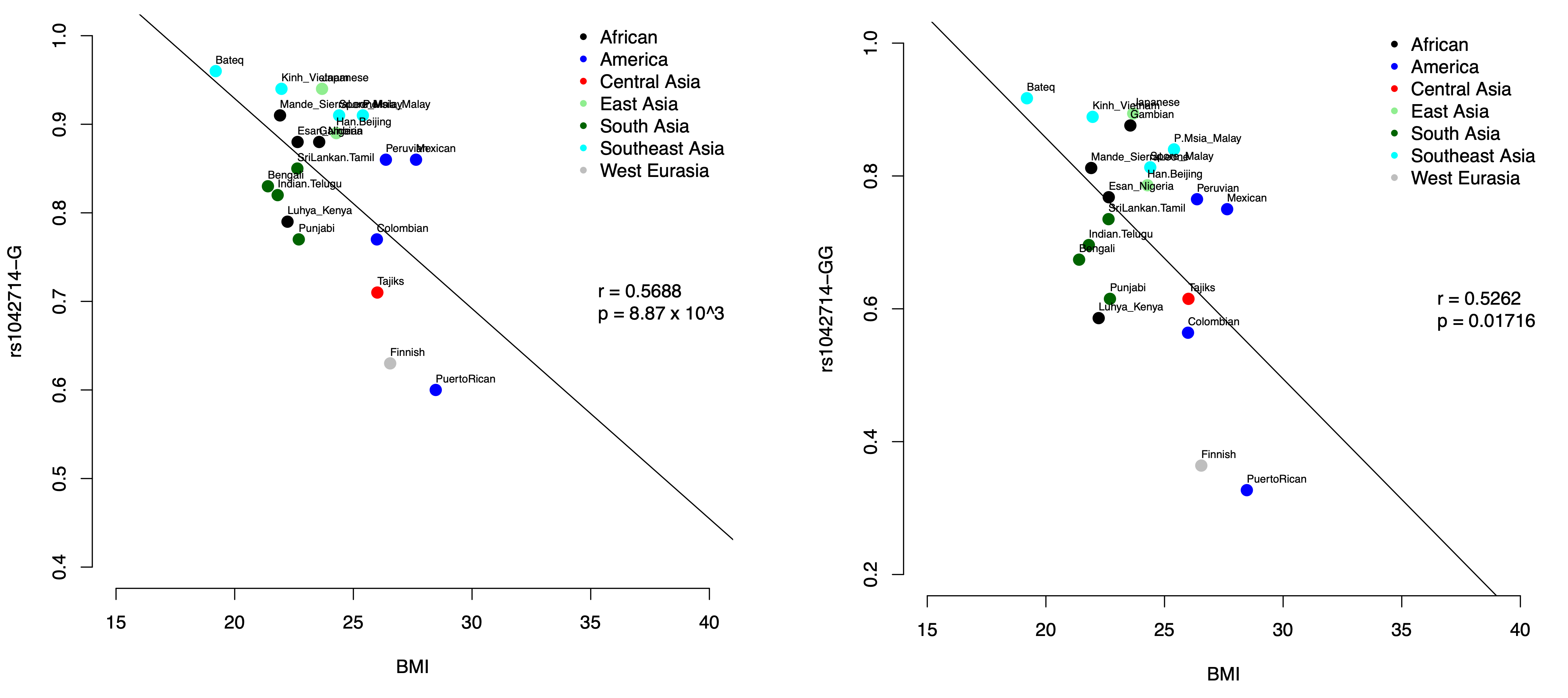
**
