# Supplementary material for: Genetic Association of the Renin-Angiotensin-Aldosterone System with hypertension among the Malays and their adaptation to climate change
Source: PLoS One. 2026 Apr 15;21(4):e0346614. doi: 10.1371/journal.pone.0346614 (PMC13082722; doi:10.1371/journal.pone.0346614)
Supplement: S7 Fig — Y axis indicates -log10(theoretical P values); X axis indicates the positions of the sliding windows. Each single panel corresponds to sliding window size. (DOCX) [file pone.0346614.s023.docx]

**S7 Fig. Tajima’s D for *AGT* and *CYP11B2* among the Malays.** Y axis indicates -log10(theoretical P values); X axis indicates the positions of the sliding windows. Each single panel corresponds to sliding window size.

**REFERENCES:**

1. Teo YY, Sim X, Ong RTH, Tan AKS, Chen J, Tantoso E, et al. Singapore Genome Variation Project: A haplotype map of three Southeast Asian populations. Genome Res. 2009;19: 2154–2162. doi:10.1101/gr.095000.109

2. Liu X, Yunus Y, Lu D, Aghakhanian F, Saw WY, Deng L, et al. Differential positive selection of malaria resistance genes in three indigenous populations of Peninsular Malaysia. Hum Genet. 2015;134: 375–392. doi:10.1007/s00439-014-1525-2

3. Yew CW, Minsong A, Tiek S, Lau Y, Pugh-kitingan J, Ransangan J, et al. Genetic relatedness of indigenous ethnic groups in northern Borneo to neighboring populations from Southeast Asia , as inferred from genome-wide SNP data. Ann Hum Genet. 2018; doi.org/10.1111/ahg.12246. doi:10.1111/ahg.12246

4. Boon-Peng H, Jusoh JAM, Marshall CR, Majid F, Danuri N, Basir F, et al. Rare copy number variants identified suggest the regulating pathways in hypertension-related left ventricular hypertrophy. PLoS One. 2016;11. doi:10.1371/journal.pone.0148755
